# Criticality enhances the multilevel reliability of stimulus responses in cortical neural networks

**DOI:** 10.1371/journal.pcbi.1009848

**Published:** 2022-01-31

**Authors:** Junhao Liang, Changsong Zhou

**Affiliations:** 1 Department of Physics, Centre for Nonlinear Studies and Beijing-Hong Kong-Singapore Joint Centre for Nonlinear and Complex Systems (Hong Kong), Institute of Computational and Theoretical Studies, Hong Kong Baptist University, Kowloon Tong, Hong Kong SAR, China; 2 Centre for Integrative Neuroscience, Eberhard Karls University of Tübingen, Tübingen, Germany; 3 Department for Sensory and Sensorimotor Systems, Max Planck Institute for Biological Cybernetics, Tübingen, Germany; 4 Department of Physics, Zhejiang University, Hangzhou, China; Porter Neuroscience Research Center, National Institute of Mental Health, UNITED STATES

## Abstract

Cortical neural networks exhibit high internal variability in spontaneous dynamic activities and they can robustly and reliably respond to external stimuli with multilevel features–from microscopic irregular spiking of neurons to macroscopic oscillatory local field potential. A comprehensive study integrating these multilevel features in spontaneous and stimulus–evoked dynamics with seemingly distinct mechanisms is still lacking. Here, we study the stimulus–response dynamics of biologically plausible excitation–inhibition (E–I) balanced networks. We confirm that networks around critical synchronous transition states can maintain strong internal variability but are sensitive to external stimuli. In this dynamical region, applying a stimulus to the network can reduce the trial-to-trial variability and shift the network oscillatory frequency while preserving the dynamical criticality. These multilevel features widely observed in different experiments cannot simultaneously occur in non-critical dynamical states. Furthermore, the dynamical mechanisms underlying these multilevel features are revealed using a semi-analytical mean-field theory that derives the macroscopic network field equations from the microscopic neuronal networks, enabling the analysis by nonlinear dynamics theory and linear noise approximation. The generic dynamical principle revealed here contributes to a more integrative understanding of neural systems and brain functions and incorporates multimodal and multilevel experimental observations. The E–I balanced neural network in combination with the effective mean-field theory can serve as a mechanistic modeling framework to study the multilevel neural dynamics underlying neural information and cognitive processes.

## Author summary

The complexity and variability of brain dynamical activity range from neuronal spiking and neural avalanches to oscillatory local field potentials of local neural circuits in both spontaneous and stimulus-evoked states. Such multilevel variable brain dynamics are functionally and behaviorally relevant and are principal components of the underlying circuit organization. To more comprehensively clarify their neural mechanisms, we use a bottom-up approach to study the stimulus–response dynamics of neural circuits. Our model assumes the following key biologically plausible components: excitation–inhibition (E–I) neuronal interaction and chemical synaptic coupling. We show that the circuits with E–I balance have a special dynamic sub-region, the critical region. Circuits around this region could account for the emergence of multilevel brain response patterns, both ongoing and stimulus-induced, observed in different experiments, including the reduction of trial-to-trial variability, effective modulation of gamma frequency, and preservation of criticality in the presence of a stimulus. We further analyze the corresponding nonlinear dynamical principles using a novel and highly generalizable semi-analytical mean-field theory. Our computational and theoretical studies explain the cross-level brain dynamical organization of spontaneous and evoked states in a more integrative manner.

## 1. Introduction

The brain is a complex system characterized by elusive ongoing and stimulus-evoked activity patterns with seemingly distinct mechanisms at different levels. To better clarify the functions and working principles of the brain, elucidating the dynamical origin of the multilevel stimulus–response relationships using neural circuits is essential.

Cortical neural systems feature high internal variability [1] across different scales. The spontaneous dynamics are high-dimensional, structured, and behavior-relevant [2]. First, the neurons function in noisy environments [3] and spike irregularly [4,5] because of the balance between the received excitatory and inhibitory current inputs [6,7]. Second, population oscillations widely occur in neural circuits within different frequency bands [8]. Third, scale-free neural avalanches (i.e., the spatial or temporal propagation of spiking activities in neuronal networks) have been widely found in vitro [9] and in vivo [10]. These neural avalanches, which satisfy critical properties [11], emerge around the oscillation transition between the asynchronous and synchronous network spiking states [12–14].

Despite the strong internal variability of cortical neural networks, they can respond to external stimuli in a fairly robust manner [15,16] and feature diverse facets. A pronounced effect of stimuli is the reduction of the trial-to-trial variability (TTV), which has been observed in the local field potential (LFP) and spiking of neurons [17] and in functional magnetic resonance imaging signals [18] across broad brain regions and species. Moreover, TTV reduction is associated with brain functioning, including cognitive and perceptual abilities [19] and brain disorders [20,21]. Another typical phenomenon is the stimulus-induced alternation in the oscillation frequency domain. For example, visual stimuli can modulate the alpha band of occipital electroencephalography signals to the beta band [22]. The gamma oscillation frequency in visual neural circuits depends on the contrast of the visual stimulus [23,24] such that a higher stimulus contrast results in a higher gamma frequency. The modulations (suppressed or enhanced) of the brain rhythm, which cover a broad spectrum (e.g., alpha (8~13Hz), gamma (25~80Hz)), have been found to be behavior-related [25–27]. Neural circuits poised at criticality are considered to have maximal dynamic ranges [28,29] and thus should most effectively respond to external stimuli. Importantly, the pre-stimulus critical properties in terms of scale-free avalanches can be preserved after the stimulus onset [30–32].

Taken together, neural systems exhibit sophisticated stimulus–response relationships, which are supported by their complex spontaneous multilevel dynamics. However, previous modeling and theoretical studies have mainly focused on specific features of spontaneous neural oscillations [33], spontaneous neural avalanches [34], or specific stimulus–response properties at certain levels in neural networks [35–37]. A mechanistic modeling approach validated by both ongoing and evoked neural dynamical features to understand such multilevel (from neuronal spiking to neural field potential) stimulus-response relations is still lacking, though such relationship has been explored in macroscopic neural field model and MEG data [37]. Recent studies [38,39] have shown that E–I balanced neural networks with suitable synaptic kinetics can reconcile multilevel complex spontaneous neural dynamics in accordance with experimental observations in terms of individual neuron spiking, population oscillation, and critical avalanches. In this paper, we further investigate the stimulus–response relationship in this type of biologically plausible neuronal networks. We find that only at the critical state around oscillation transition can the circuit reproduce the experimental observation of external stimulus–induced TTV reduction, effectively modulate the gamma frequency, and preserve the circuit critical properties. Based on a novel semi-analytical mean-field theory [39], we elucidate the dynamical mechanism behind such multilevel features. Specifically, spiking neural networks with critical dynamics near an oscillation transition correspond to macroscopic field equations poised near the Hopf bifurcation. Increasing stimulus strength modulates (increases) the Hopf frequency but has little influence on the Hopf stability of the equilibria in the field equations; consequently, in spiking networks, the gamma frequency is modulated (increased), but the criticality is preserved. Linear approximation analysis reveals that fluctuations around the equilibria near the bifurcation point are larger than those far from bifurcation and more suppressible by extra stimuli, which explains why networks at critical states show stronger internal variability but a more pronounced TTV reduction in the presence of stimuli. In summary, our study reveals the generic, integrative dynamical principles of the multilevel stimulus–response phenomena of biological neural networks found in different experiments. The neural network model and mean-field theory here can serve as a biologically plausible modeling framework to study the multilevel stimulus–response dynamics and their relationship with cognition, brain function, and brain disorders.

## 2. Methods

### 2.1 Spiking neuronal network

We study a widely investigated biologically plausible conductance-based excitation–inhibition (E–I) neural circuit [40] with an *N*_*E*_-to-*N*_*I*_ ratio of 4:1, where *N*_*E*_ is the number of excitatory neurons and *N*_*I*_ is the number of inhibitory neurons. Unless specified otherwise, we consider the default network size *N* as 2500, where *N* = *N*_*E*_+*N*_*I*_. To examine the robustness of the model dynamics and especially the properties of critical avalanches, we consider additional simulations using larger networks, with *N* = 5000, 10,000, 15,000. The neurons are connected randomly, with probability *p* = 0.2, mimicking a small local cortical column. For the *i*-th neuron, we denote its spiking train as $s_{i}(t)=\sum_{n}\delta (t-t_{i}^{n})$; its *α* (*α* can be excitatory or inhibitory) neighbors as $C_{i}^{\alpha }$; its membrane potential (voltage) as *V*_*i*_(*t*); and its input conductance (synaptic time course) received from recurrent excitatory, recurrent inhibitory, and external excitatory neurons as *GE*_*i*_(*t*), *GI*_*i*_(*t*), and *GO*_*i*_(*t*), respectively. The dynamic equations of the conductance-based leaky integrate-and-fire (IF) network can be written as

$$\frac{dV_{i}}{dt}=\frac{V_{rest}^{\alpha }-V_{i}}{\tau_{\alpha }}+(V_{E}^{rev}-V_{i})[g_{\alpha o}GO_{i}(t)+g_{\alpha E}GE_{i}(t)]+(V_{I}^{rev}-V_{i})g_{\alpha I}GI_{i}(t).$$ (1)

Here, Eq (1) describes the membrane potential evolution of the *i*-th neuron belonging to class *α*∈{*E*,*I*}. The reversal potential for excitatory and inhibitory synaptic currents are $V_{E}^{rev}=0\;mV$ and $V_{I}^{rev}=-70\;mV$, respectively. Following previously justified parameter values [38], the synaptic strengths of conductance are set as $g_{EO}=\frac{{\hat{g}}_{EO}}{\sqrt{N}}$, $g_{IO}=\frac{{\hat{g}}_{IO}}{\sqrt{N}}$, $g_{EE}=\frac{{\hat{g}}_{EE}}{\sqrt{N}}$, $g_{IE}=\frac{{\hat{g}}_{IE}}{\sqrt{N}}$, $g_{EI}=\frac{{\hat{g}}_{EI}}{\sqrt{N}}$, $g_{II}=\frac{{\hat{g}}_{II}}{\sqrt{N}}$, with ${\hat{g}}_{EO}=2.5$, ${\hat{g}}_{IO}=4$, ${\hat{g}}_{EE}=2$, ${\hat{g}}_{IE}=4$, ${\hat{g}}_{EI}=27$, ${\hat{g}}_{II}=48$. Here, the synaptic strengths scale with the network size as $∼\frac{1}{\sqrt{N}}$. This is a feature of the E-I balanced network [41] that enables systematic simulations for networks with different sizes while maintaining the E–I balance. The first term of Eq (1) describes the leaky current, which causes the membrane potential to drop back to the leaky potentials, which are set as $V_{rest}^{E}=V_{rest}^{I}=-70\;mV$. The membrane time constants are set as *τ*_*E*_ = 20 *ms*, *τ*_*I*_ = 10 *ms*. The other terms in Eq (1) represent the received excitatory and inhibitory currents of the neuron. The recurrent network input conductances are the summations of the filtered pulse trains $GE_{i}(t)=\sum_{j\in C_{i}^{E}}F^{E}*s_{j}(t)$ and $GI_{i}(t)=\sum_{j\in C_{i}^{I}}F^{I}*s_{j}(t)$. Here, the synaptic filter is modeled as an exponential function:

$$F^{\alpha }(t)=\frac{1}{\tau_{d}^{\alpha }}\exp\left(-\frac{t}{\tau_{d}^{\alpha }}\right),\;t\geq 0.$$ (2)

This function models the non-instant transmission process of neurotransmitters following the presynaptic spikes. The synaptic decay times $\tau_{d}^{E},\;\tau_{d}^{I}$ depend on the type of presynaptic neuron. We set $\tau_{d}^{E}=4\;ms$, and the value of $\tau_{d}^{I}$ varies in the range of 3~14 *ms*, which allows the network to exhibit different dynamic modes. Hence, $\tau_{d}^{I}$ serves as a control parameter to tune different background dynamic regions. The values of the synaptic decay times $\tau_{d}^{E}$, $\tau_{d}^{I}$ biologically depend on the constitution of synaptic receptors. The $\tau_{d}^{E}$ and $\tau_{d}^{I}$ values adopted in this study are close to the synaptic decay times of the AMPA and GABAa receptors [42,43] for excitation and inhibition synapses, respectively.

**Fig 1:**
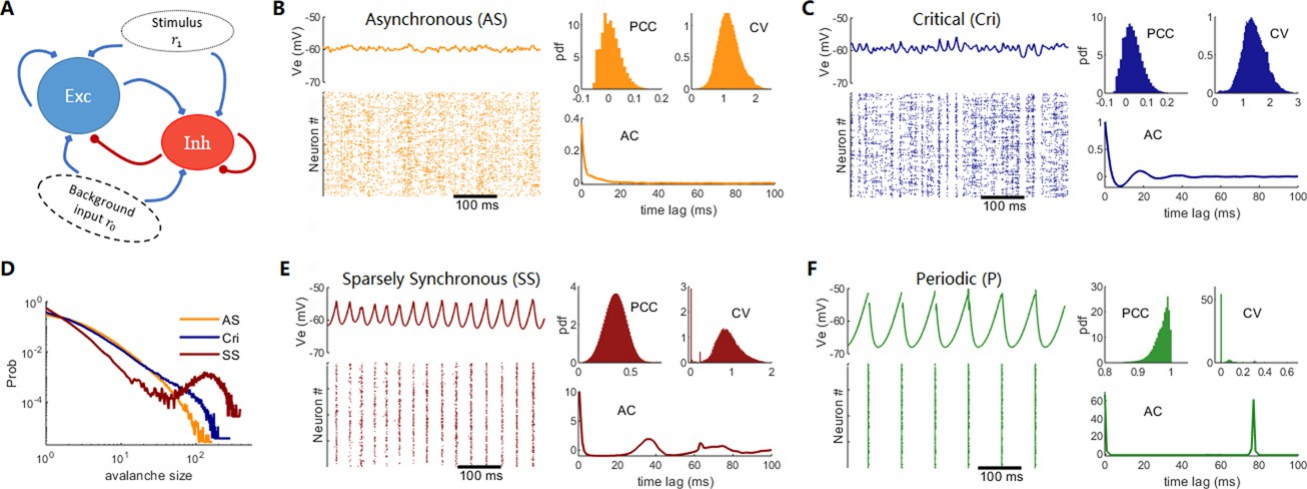
Model architecture and dynamic modes: **(A)** Diagram of the recurrent excitation–inhibition network. External input *r*_*ext*_ has a background part *r*_0_ and an extra stimulus part *r*_1_. **(B, C, E, F)** Examples of dynamic modes for AS, Cri, SS, and P states. In each case, a period of the local field potential and the corresponding spike raster plot of Exc neurons, the distributions of the Pearson correlation coefficient (PCC) and coefficient of variance (CV) of inter-spike intervals, and the population activity autocorrelation (AC) are shown. **(D)** Examples of avalanche size distributions for AS, Cri, and SS states. Here, the background input strength is *r*_0_ = 0.8/*ms*, and synaptic parameters are $\tau_{d}^{I}=4,\;8,\;11,\;14ms$ for AS, Cri, SS, and P states, respectively.

**Fig 2:**
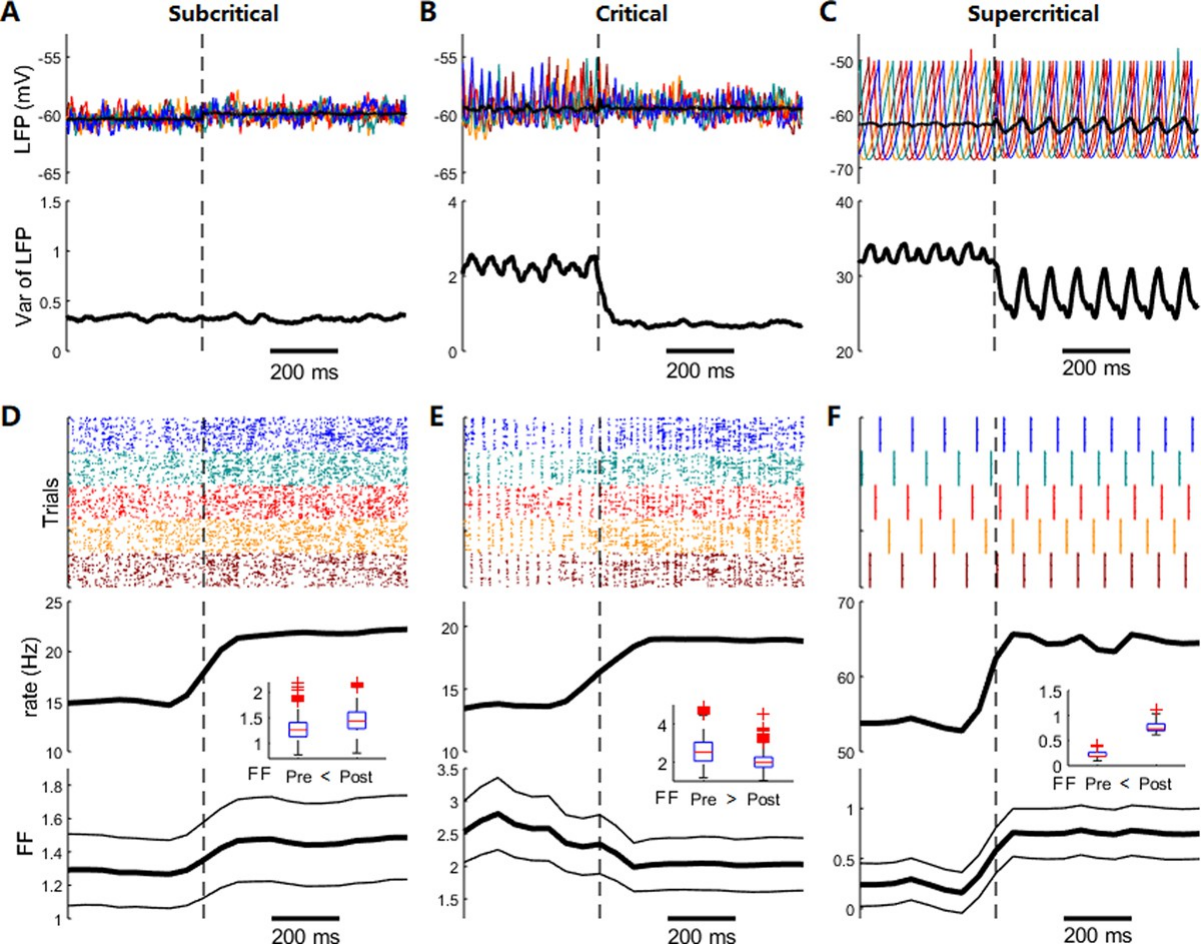
**Trial-to-trial variability in ongoing and stimulus states: (A–C)** Upper panels: the local field potentials (LFPs) of five single trials (labeled in different colors) and the all-trial-averaged LFP (bold black line). Lower panels: the cross-trial variance of LFP. **(D–F)** Upper panels: raster plots of 300 Exc neurons in five trials (labeled in different colors). Lower panels: trial-averaged firing rate and Fano factor (FF) (flanking traces are the value of ±std, where the std is due to the different sampling neuron groups used in the FF computation). The insets compare the pre- and post-stimulus FF using boxplots. Dashed black vertical lines indicate the stimulus onset time. The results of subcritical (left), critical (middle), and supercritical (right) dynamics are for parameters $\tau_{d}^{I}=4,\;8,\;and\;14\;ms$, respectively. The background input rate is *r*_0_ = 0.55/*ms*, and the stimulus strength is *r*_1_ = 0.2/*ms*.

**Fig 3:**
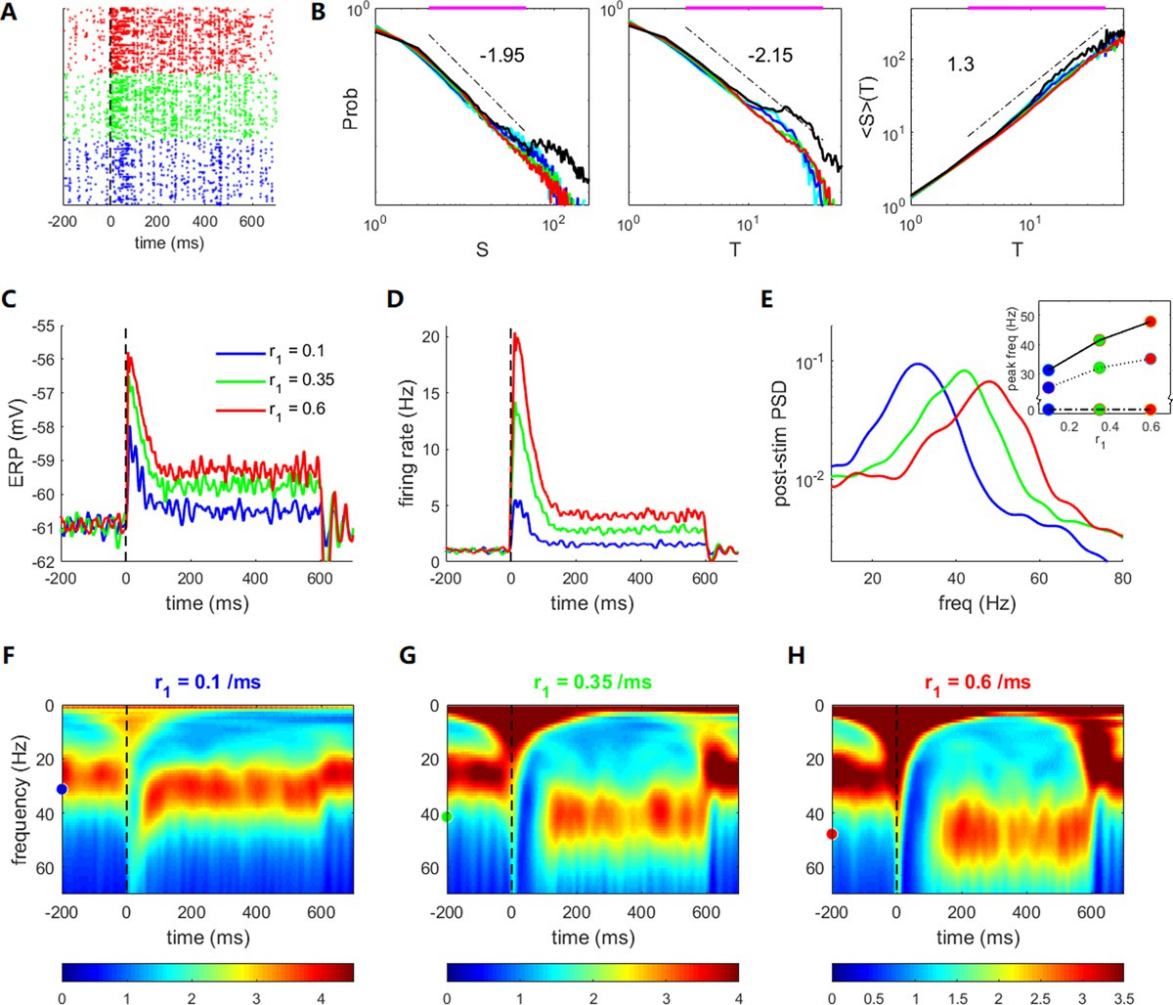
Event-related potential (ERP), gamma frequency modulation, and criticality preservation induced by stimuli with different strengths. Stimulus starts at *t* = 0 and ends at *t* = 600 *ms*. The blue, green, and red colors in **(A–E)** represent post-stimulus strengths *r*_1_ = 0.1, 0.35, *and* 0.6/*ms*, respectively. **(A)** Raster plots of 500 neurons in a trial. **(B)** Distributions of avalanche size *S*, avalanche duration *T*, and average size 〈*S*〉 for duration *T* in the post-stimulus period. The top horizontal purple lines indicate the ranges of estimated power-law distributions for the case of *r*_1_ = 0.35/*ms*. For comparison, we also show the avalanche distributions in the pre-stimulus period (cyan curves) and transient period within 100 ms after the stimulus onset (black curves) for the case of *r*_1_ = 0.1/*ms*. These properties are similar to those for other stimulus strengths *r*_1_. **(C)** ERPs under different input strengths. **(D)** Transient network firing rates. **(E)** Power spectrum density (PSD) of post-stimulus local field potential (LFP), measured 100–600 ms after stimulus onset. The inset shows the peak frequencies for different input strengths (dashed-dotted, solid, and dotted lines represent the subcritical, critical, and supercritical cases, with $\tau_{d}^{I}=5,\;10,\;and\;13\;ms$, respectively). **(F–H)** Time evolution of the powers of different LFP oscillation frequencies for different input strengths. Dots at *t* = −200 *ms* indicate the post-stimulus peak frequency. The background input is *r*_0_ = 0.3/*ms*. The synaptic parameter is $\tau_{d}^{I}=9\;ms$ for **(A, B)** and $\tau_{d}^{I}=10\;ms$ for **(C–H)**.

**Fig 4:**
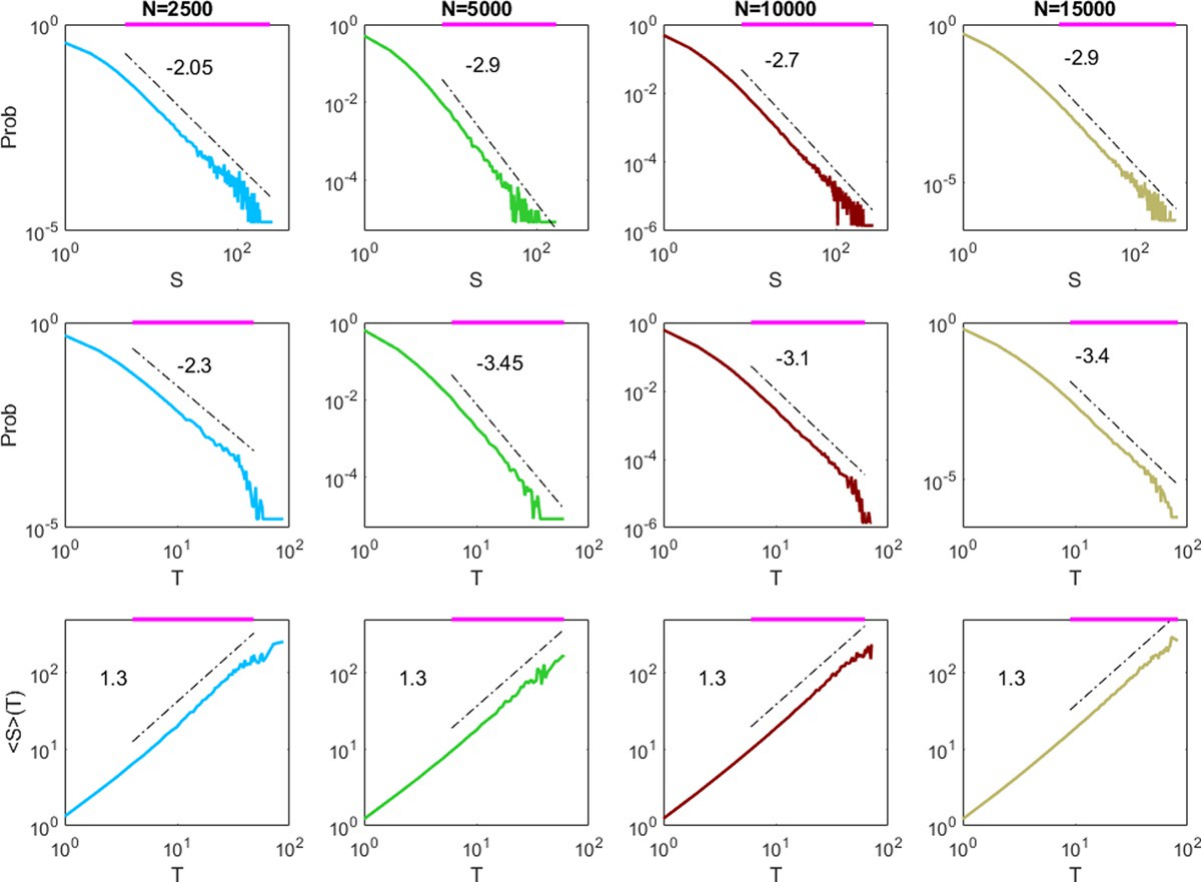
Critical avalanches in networks with different sizes. We simulate networks subjected to noisy inputs with a constant strength *r*_*in*_ that linearly scales with the network size and exhibits critical dynamics ($\tau_{d}^{I}=9\;ms$). We show the distributions of avalanche size *S*, avalanche duration *T*, and the average size 〈*S*〉 under duration *T*. Horizontal purple lines indicate the ranges of estimated power-law distributions. From left to right, the network sizes are *N* = 2500, 5000, 10,000, 15,000. The input strengths are *r*_*in*_ = 0.9, 1.8, 3.6, 5.4/*ms*, to maintain the scale condition *r*_*in*_~*O*(*N*) for E–I balanced networks. Avalanches are measured with adapted time bin Δ*t* = *T*_*m*_ = 0.11, 0.03, 0.02, 0.013 *ms* respectively.

For the external input, we consider two scenarios. The first case is the deterministic input *GO*_*i*_(*t*) = *r*_*in*_(*t*) (for results in Figs 1 and 2), and the second case is the noisy input $GO_{i}(t)=F^{E}*\sum_{n}\delta (t-T_{i}^{n})$, which is modeled as the filtered spike train ${\left\{T_{i}^{n}\right\}}_{n\geq 1}$ from a time-heterogeneous Poisson process with time-varying rate *r*_*in*_(*t*) (for results in Figs 3 and 4). In both cases, the input rate *r*_*in*_(*t*) can be decomposed as

$$r_{in}(t){=r}_{0}+r_{ext}(t).$$ (3)

Here, *r*_0_ is the background input rate of the network and is constant over time, and *r*_*ext*_(*t*) is the extra stimulus applied to the network, starting from time *t*_*onset*_ and with stimulus strength *r*_1_. Sensory circuits generally adapt [44] to a change in the input strength such that for a sudden increase in the external input, the system response is first intense and then mitigated, causing a waveform termed an event-related potential (ERP) [45] in some detected signals. In neural networks, such an adaptation mechanism may be modeled by plasticity rules such as short-term depression [31]. However, our model does not include an adaptation mechanism. Thus, in the simulations in Fig 3, to mimic the ERP-causing effect, we adopt the input rate as

$$r_{ext}(t)=\left\{ \begin{array}{c} r_{1}+a(t-t_{onset})e^{-(t-t_{onset})/\tau_{0}},\;t\geq t_{onset}\\ 0,\;t<t_{onset} \end{array}\right..$$ (4)

This rate represents a step-increase plus a transient pulse modeled as an alpha function, with the pulse strength *a* = *r*_1_/*ms*; that is, a higher step-increase will result in a stronger transient pulse. The maximum instantaneous rate *r*_*m*_ = *aτ*_0_/*e* is reached at time *t* = *τ*_0_. Experiments have shown that rats process olfactory sensory signals in 200 ms [46], and the human visual system can process signals in 150 ms [47]. Thus, a reasonable *τ*_0_ value is 10 to 100 ms, and we set *τ*_0_ = 20 ms. When studying TTV, we use deterministic inputs, with *r*_*ext*_(*t*) given by Eq (4), where *a* = 0 (i.e., no transient pulse). Moreover, the network firing rates under deterministic and stochastic inputs are almost the same. Note that we assume a fixed connection probability *p* = 0.2 in the network (representing dense connectivity [41]); therefore, the neighbors of a neuron and the recurrent input that a neuron receives increase with increasing network size. Another modeling assumption of such dense E–I balanced networks [41] is that the external inputs have the same scale as the recurrent inputs. Thus, the strength of the external input (*r*_*in*_ in the following) should scale with ~*N*. For simplicity, we do not explicitly include this dependence in the formula of *r*_*in*_, but we will maintain the scaling relationship when comparing the results of networks with different sizes. For example, the strength of the external input of a network with *N* = 5000 should be twice that of a network with *N* = 2500.

The network dynamics are simulated using a modified second-order Runge–Kutta scheme [48], with a time step of *dt* = 0.05 *ms*. When the membrane potential reaches the threshold *V*_*th*_ = −50 *mV*, a spike is emitted, and the membrane potential is reset to *V*_*reset*_ = −60 *mV*. Then, the synaptic integration is halted for 2 ms for excitatory neurons and 1 ms for inhibitory neurons, modeling the refractory periods in real neurons.

### 2.2 Mean-field theory of the spontaneous and response dynamics of spiking neuronal network

To understand the dynamical mechanism of the network properties and their stimulus-induced modulation, we derive the macroscopic field equation corresponding to the spiking neuronal network model (Eq 1) using a novel semi-analytical mean-field theory [39]. Comprehensive details of the mean-field derivation can be found in [39]. In [39], the derivation was mainly performed in a current-based model, while the derivation of the conductance-based model (similar to the model studied in the current paper) was included in the supplementary material of [39].

Let *V*_*E*_ = 〈*V*_*i*_〉_*i*∈*E*_ and *V*_*I*_ = 〈*V*_*i*_〉_*i*∈*I*_ represent the average excitatory and inhibitory voltages, respectively, and Φ_*E*_ = 〈*GE*_*i*_〉_*i*∈*E or I*_ and Φ_*I*_ = 〈*GI*_*i*_〉_*i*∈*E or I*_ represent the excitatory and inhibitory received recurrent input conductances of the network, respectively. We are most interested in the network properties with specific synaptic decay time $\tau_{d}^{I}$ and input strength. Thus, we consider the case where the external input is a constant deterministic value: *GO*_*i*_(*t*) = *r*_*in*_. Then, introducing the population average 〈∙〉_*i*∈*E*_ and 〈∙〉_*i*∈*I*_ into Eq (1) and performing the decoupling approximation 〈[*GE*_*i*_+*GI*_*i*_]*V*_*i*_〉_*i*∈*α*_≈〈*GE*_*i*_+*GI*_*i*_〉_*i*∈*α*_〈*V*_*i*_〉_*i*∈*α*_, we get

$$\frac{dV_{\alpha }}{dt}=\frac{V_{rest}^{\alpha }-V_{\alpha }}{\tau_{\alpha }}+[g_{\alpha O}r_{in}+{g_{\alpha E}\Phi }_{E}](V_{E}^{rev}-V_{\alpha })+{g_{\alpha I}\Phi }_{I}(V_{I}^{rev}-V_{\alpha }),\;\alpha =E,I.$$ (5)

The firing rate of the *α* neurons at time *t* (denoted as *Q*_*α*_(*t*)), defined as the proportion of neurons whose membrane potential *V*_*i*_ is above the spiking threshold *V*_*th*_ (before the resetting rule applies), can be approximately computed by assuming a Gaussian distribution of the membrane potential with mean *V*_*α*_ and standard deviation *σ*_*α*_ [39].


$$Q_{\alpha }(t)={\langle \sum_{n}\delta (t-t_{i}^{n})\rangle }_{i\in \alpha }=1/[1+exp(\frac{V_{th}-V_{\alpha }}{\sigma_{\alpha }}\frac{\pi }{\sqrt{3}})],\;\alpha =E,I.$$ (6)

Here, *Q*_*α*_(*t*) represents the proportion of *α*-type neurons that spike between *t* and *t*+Δ*t* (Δ*t* is an infinitely small quantity). It also denotes the mean firing rate per ms of *α-*type neurons at time *t* [39]. We aim to obtain field equations that can effectively capture the desired nonlinear dynamic properties of the spiking network, so that the network response properties can be understood through the nonlinear dynamic analysis of the field equations. Here, *σ*_*α*_, representing the standard deviation of the membrane potential of neural population *α*, is an effective parameter to construct the voltage-dependent mean population firing rate Eq (6). It cannot be analytically derived and should be estimated numerically. Although the optimal *σ*_*α*_ values may depend on the input strength *r*_*in*_, we choose the effective parameters as *σ*_*E*_ = 3.2 and *σ*_*I*_ = 3.8 (suitable values obtained from numerical tests). With these values, the derived field equation can quantitatively capture several essential features of the neuronal network (see details in Section 3.4). The dependence of the predicted bifurcation value on these effective parameters is further studied (S9 Fig). However, a comprehensive analytical approach for studying the dynamics of conductance-based IF neural networks remains an open issue [49,50].

Assuming that the total *α* inputs of each neuron is the same (mean-field approximation), we have ${\langle \sum_{j\in C_{i}^{\alpha }}s_{j}(t)\rangle }_{i\in E\;or\;I}=n_{\alpha }Q_{\alpha }(t)$, where *n*_*α*_ = *pN*_*α*_ is the average number of *α* neighbors of a neuron in the network. The convolution *F*^α^*δ(*t*), where *F*^*α*^ is given by Eq (2), obeys $\left(\tau_{d}^{\alpha }\frac{d}{dt}+1)[F^{\alpha }*\delta (t)]=\delta (t\right)$, so that $(\tau_{d}^{\alpha }\frac{d}{dt}+1)[\sum_{j\in C_{i}^{\alpha }}F^{\alpha }*s_{j}(t)]=\sum_{j\in C_{i}^{\alpha }}s_{j}(t)$. Taking the population average 〈∙〉_*i*∈*E or I*_, we have $\left(\tau_{d}^{\alpha }\frac{d}{dt}+1)\Phi_{\alpha }=n_{\alpha }Q_{\alpha }(t\right)$; that is,

$$\tau_{d}^{\alpha }\frac{d\Phi_{\alpha }}{dt}=-\Phi_{\alpha }+{pN}_{\alpha }/\left[1+\exp\left(\frac{V_{th}-V_{\alpha }}{\sigma_{\alpha }}\frac{\pi }{\sqrt{3}}\right)\right],\;\alpha =E,I.$$ (7)

Eqs (5) and (7) are the deterministic field equations to study the dynamic properties of the spiking network, whose dynamic is represented by Eq (1).

To further explore the dynamic fluctuation and TTV property of the spiking network from field equations, we introduce artificial noise sources $\sqrt{\beta }\xi_{\alpha }(t),$ *α* = *E*,*I* into the membrane potential equation Eq (5), where *ξ*_*E*_(*t*) and *ξ*_*I*_(*t*) are the independent standard (with zero mean and unit variance) Gaussian white noises (GWNs), and *β* indicates the noise strength. The GWN in field equations is phenomenological, and *β* does not truly reflect the noise level in the spiking network.

In summary, the noisy field equations corresponding to the spiking network take the form

$$\left\{ \begin{array}{c} \frac{dV_{\alpha }}{dt}=\frac{V_{rest}^{\alpha }-V_{\alpha }}{\tau_{\alpha }}+[g_{\alpha O}r_{in}(t)+g_{\alpha E}\Phi_{E}](V_{E}^{rev}-V_{\alpha })+g_{\alpha I}\Phi_{I}(V_{I}^{rev}-V_{\alpha })+\sqrt{\beta }\xi_{\alpha }(t)\\ \tau_{d}^{\alpha }\frac{d\Phi_{\alpha }}{dt}=-\Phi_{\alpha }+n_{\alpha }/[1+\exp\left(\frac{V_{th}-V_{\alpha }}{\sigma_{\alpha }}\frac{\pi }{\sqrt{3}}\right)],\;\alpha =E,\;I \end{array}\right..$$ (8)

We can analyze the stability of the equilibrium and the strength of noise fluctuation around the equilibrium of Eq (8).

The deterministic equilibrium (fixed point) of the field equations Eq (8) is found by setting *d*/*dt* = 0 and *β* = 0, resulting in algebraic equations.


$$\frac{V_{rest}-V_{\alpha }}{\tau_{\alpha }}+[g_{\alpha O}r_{in}+g_{\alpha E}Q_{E}](V_{E}^{rev}-V_{\alpha })+g_{\alpha I}Q_{I}(V_{I}^{rev}-V_{\alpha })=0,\;\alpha =E,I.$$ (9)

Here, *Q*_*α*_ depends on *V*_*α*_ through the relationship in Eq (6). The equilibrium value does not depend on $\tau_{d}^{\alpha }$ because the synaptic filter is normalized ($\int_{0}^{\infty }F^{\alpha }*\delta (t)=1$ independent of $\tau_{d}^{\alpha }$), while the equilibrium stability depends on $\tau_{d}^{\alpha }$. The Jacobian matrix of Eq (8) at the equilibrium is

$$J=[\begin{array}{cccc} -\frac{1}{\tau_{E}}-(g_{EO}r_{in}+g_{EE}\Phi_{E}+g_{EI}\Phi_{I}) & 0 & (V_{E}^{rev}-V_{E})g_{EE} & (V_{I}^{rev}-V_{E})g_{EI}\\ 0 & -\frac{1}{\tau_{I}}-(g_{IO}r_{in}+g_{IE}\Phi_{E}+g_{II}\Phi_{I}) & (V_{E}^{rev}-V_{I})g_{IE} & (V_{I}^{rev}-V_{I})g_{II}\\ \frac{n_{E}Q_{E}^{\prime }(V_{E})}{\tau_{d}^{E}} & 0 & -\frac{1}{\tau_{d}^{E}} & 0\\ 0 & \frac{n_{I}Q_{I}^{\prime }(V_{I})}{\tau_{d}^{I}} & 0 & -\frac{1}{\tau_{d}^{I}} \end{array}],$$ (10)

with $Q_{\alpha }^{\prime }(V_{\alpha })=\frac{\pi exp[(V_{th}-V_{\alpha })\pi /(\sqrt{3}\sigma_{\alpha })]}{\sqrt{3}\sigma_{\alpha }{\left(1+\exp\left[\frac{(V_{th}-V_{\alpha })\pi }{\sqrt{3}\sigma_{\alpha }}\right]\right)}^{2}}$ estimated at the steady-state value of *V*_*α*_ given by Eq (9). The eigenvalues of *J* can determine the stability of the steady state.

Furthermore, we evaluate the fluctuation around the equilibrium of Eq (8) using the linear noise approximation (LNA) method [51]. Denote *X* = (*V*_*E*_, *V*_*I*_, Φ_*E*_, Φ_*I*_)^*T*^ as the state variables of Eq (8). The linearized equation at the equilibrium is

$$\frac{dX}{dt}=JX+\sqrt{B}\xi (t).$$ (11)

Here, *ξ*(*t*) = (*ξ*_*E*_(*t*),*ξ*_*I*_(*t*),0,0)^*T*^ and *B* = *diag*(*β*,*β*,0,0) is the noise covariance matrix (notation *diag* represents diagonal matrix). Moreover, Σ = *cov*(*X*,*X*) represents the covariance matrix of the state variable *X*, and it obeys [51]

$$\frac{d\Sigma }{dt}=J\Sigma +\Sigma J^{T}+B.$$ (12)

Thus, the covariance at the stationary state can be computed by numerically solving the Lyapunov equation

$$J\Sigma +\Sigma J^{T}+B=0.$$ (13)

The fluctuation around the equilibrium is given by the diagonal elements of Σ. For example, the first element of Σ is the variance of *V*_*E*_. We denote it as *Var*(*V*_*E*_), and it depends on the external input strength *r*_*in*_. These dynamic fluctuations can effectively approximate the TTV of the spiking neural network.

### 2.3 Statistical analysis

#### 2.3.1 Local field potential

The LFP is a common measure of neuronal activity. It is, however, not completely clear how the LFP is related to single-neuron variables such as synaptic or ionic currents and membrane potential [52]. It is likely that LFPs were originated in synaptic currents on pyramidal neuron dendrites [53]. In general, such dendrite properties are not considered in our point model as it lacks a spatial geometric structure of neurons; thus, an approximation is needed. Mazzoni et al. [54] studied different approximation schemes to compute the LFP in point IF neuron models, including factors about membrane potentials and synaptic currents. They found that a model-specific time-delayed linear combination of AMPA and GABAa currents constitutes a best approximation of LFP, while the average membrane potential of neurons, as commonly used in many models [55], is a fair choice to achieve a reasonable approximation. Moreover, it was shown that the average membrane potential as LFP is qualified in terms of information content [35]. Taken together, we simply take the average membrane potential of the excitatory neurons in the network, *V*_*e*_(*t*) = 〈*V*_*i*_(*t*)〉_*i*∈*E*_, as the LFP. Separately, we also examine the effect of inhibitory neurons by defining the LFP as the average membrane potential of the inhibitory neurons or all of the neurons in the network. None of these definitions result in any essential difference in the LFP properties (see S1 Fig). We apply wavelet analysis to the single-trial LFP with a complex Morlet wavelet basis to obtain the patterns of frequency components (the absolute value of the wavelet coefficients) across time. The presented pattern is obtained by averaging the wavelet coefficients of all of the trials (in Figs 3 and S7).

#### 2.3.2 Trial-to-trial variability quantification

The TTV in our model can arise from different realizations of random network topology, different initial membrane potentials of neurons, and noisy inputs. Here, we study the intrinsic variability caused by the network dynamic nature of E–I balance and criticality. Thus, in our study of TTV, we apply a constant input *GO*_*i*_(*t*) = *r*_0_+Θ(*t*−*t*_*onset*_) *r*_1_ to the network (Θ(*x*) is the Heaviside step function). For each parameter setting, we first set up a fixed randomly generated network topology. Then, we simulate the network dynamics under 100 different initial conditions. The initial membrane potential *V*_*initial*_ of each neuron is generated from a normal distribution, with mean *m* and standard deviation *σ*. For each trial, *m* and *σ* are determined from uniform distributions in [−70, −55] and [0, 5], respectively (if a generation *V*_*initial*_ is greater than −50, then it is replaced by another value uniformly distributed from [−70, − 50].) The stimulus onset time *t*_*onset*_ is equal to 1 s.

a. The TTV of LFP. We use the cross-trial variance of LFP to quantify its TTV. The LFP is measured in 1 ms resolution, and the cross-trial variance (time series) is then computed. The plot of LFP versus time (in Fig 2) is smoothed using a 30 ms square window.

b. The TTV of spiking. We use the commonly used cross-trial Fano factor (FF) [56] of the neuron spike numbers to quantify the spiking TTV in time scale ~100ms, as in the analysis of TTV of LFP and neuron spiking in monkey and cat cortices [17]. FF estimation is often problematic [56], especially in the cases of low firing rates or short recording time. Thus, we compute the FF using the merged spike train of *n* neurons (i.e., a combination of *n* neurons represents one measurement unit) to avoid the ill behavior resulting from too few spikes. This spike train also mimics the measured multiunit activity of detected spikes of multiple neurons close to an electrode in experiments. The FF is computed by the spike number series of this neuron group in every 100 ms window, with a step of 50 ms. Here, *n* out of 2000 excitatory neurons are randomly sampled to compute the FF series, and the results of 200 samples are averaged. We use *n* = 5, and the results are broadly unchanged when *n*>5 (S2 Fig). The plot of FF versus time (Figs 2 and S2) is smoothed by a square window with length of 5 moving steps, namely length 250 ms. The pre-stimulus and post-stimulus variance/FF values (Fig 2) correspond to the ranges from −550 *ms* to −50 *ms* and from 50 *ms* to 550 *ms*, respectively (when 0 *ms* is taken as the stimulus onset time). Here, we use the raw FF rather than the mean-match FF [17]. The mean-match procedure requires choosing a group of recording units that preserve the mean spike count distributions across time, which cannot be implemented in certain dynamic regions of our model. Although the mean-match method has often been used to control the firing rate to better compare the pre- and post-stimulus FF, the results of the raw FF and mean-match FF have been shown to be similar in most cases [17].

#### 2.3.3 Spike train statistics

The Pearson correlation coefficient (PCC) between neurons *i* and *j* is defined as $c_{ij}=\frac{cov({\tilde{N}}_{i}(t),{\tilde{N}}_{j}(t))}{\sqrt{var({\tilde{N}}_{i}(t))var({\tilde{N}}_{j}(t))}}$, where ${\tilde{N}}_{i}(t)$ is the spike series of neuron *i* filtered by a square kernel with length *T* = 5 *ms* that ${\tilde{N}}_{i}(t)=\sum_{s=0}^{T-1}N_{i}(t-s)$, where *N*_*i*_(*t*) is the spike count series constructed with time window Δ*t* = 1 *ms*. The autocorrelation (AC) of the population activity is defined as $AC(\tau )=\frac{1}{n_{0}^{2}T}\sum_{t=1}^{T}\left(n(t+\tau )-n_{0})(n(t)-n_{0}\right)$, where *n*(*t*) = ∑_*i*_*N*_*i*_(*t*) is the population activity (number of spikes) in every 1 ms, and *n*_0_ is the average *n*(*t*) value. To measure the spiking time irregularity, we also calculate the coefficient of variance (CV) of inter-spike intervals (ISIs). The PCC, CV, and AC results (Fig 1) are obtained from the average results of 200 trials of 1 s simulation. For the PCC and CV distributions, only neurons with ≥ 5 spikes are selected for analysis.

#### 2.3.4 Neuronal avalanches and critical property

**Fig 5:**
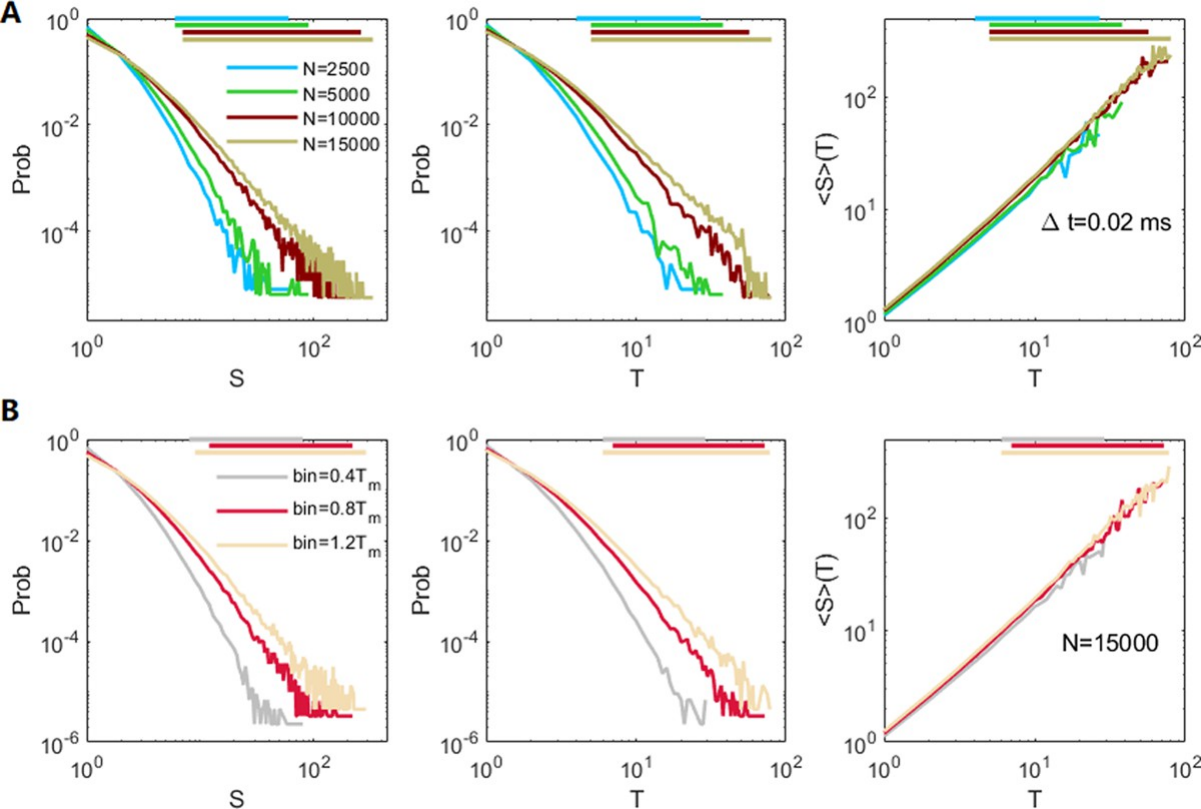
Further measurement of critical avalanches using different sizes of time bins. We further measure avalanches in networks in Fig 4 using different time bins Δ*t*. The distributions of avalanche size *S*, avalanche duration *T*, and the average size 〈*S*〉 under duration *T* are shown. **(A)** Measurement of avalanches in networks with sizes *N* = 2500, 5000, 10,000, 15,000 using fixed bin Δ*t* = 0.02*ms*. **(B)** Measurement of avalanches in a network with size *N* = 15,000 using different time bins Δ*t* = 0.4*T*_*m*_, 0.8*T*_*m*_ and 1.2*T*_*m*_, where *T*_*m*_ = 0.013 *ms*. The horizontal lines on top indicate the ranges of estimated power-law distributions of the corresponding cases.

Following a recent observation that the irregular spiking of pyramidal neurons in vivo exhibits scale-free features [57], we measure the neuronal avalanches in the excitatory neuronal population. We count the spike numbers in each window (bin) with length Δ*t* for the merged spike trains of excitatory neurons. An avalanche is defined as a sequence of consecutive spiking periods (bins), separated by a silent period. The size *S* of an avalanche is defined as the total number of spikes within the period, and the duration *T* is defined as the number of time bins it contains. In general, we choose the average ISI of the merged spiking train (denoted as *T*_*m*_,) as the bin length Δ*t* [9], which depends on the dataset property. The choice of Δ*t* is crucial for identifying the avalanche properties. In Fig 3, for the critical dynamic region with $\tau_{d}^{I}=9\;ms$, the time bin is Δ*t* = *T*_*m*_ = 0.45 *ms* in the pre-stimulus spontaneous period with *r*_0_ = 0.3/*ms*. In the post-stimulus periods with strengths *r*_1_ = 0.1, 0.35, 0.6/*ms*, the adapted time bins are Δ*t* = *T*_*m*_ = 0.29, 0.15, 0.11 *ms*, respectively. In Fig 4, for networks with *N* = 2500, 5000, 10,000, and 15,000, the adapted time bins are Δ*t* = *T*_*m*_ = 0.11, 0.03, 0.02, 0.013 *ms*, respectively, decreasing with network size. We also measure avalanches with other choices of time bins Δ*t*≠*T*_*m*_ in Fig 5.

The avalanche size and duration distributions are first inspected visually. For distributions appear to follow the power law, we use a doubly truncated algorithm based on the maximum likelihood estimation in the NCC toolbox [58] to find the ranges that pass the truncation-based Kolmogorov–Smirnov (K–S) statistics test (with *p* values larger than 0.1) and estimate the critical exponents. The estimated slopes within the truncated ranges in the avalanche size and duration distributions define the critical exponents *P*(*S*)~*S*^−*τ*^ and *P*(*T*)~*T*^−*α*^. A third exponent is defined as 〈*S*〉(*T*)~*T*^1/*συz*^, where 〈*S*〉(*T*) is the average size of avalanches with the same duration *T*, and it is fitted using a weighted least squares method [58]. Scaling relation $\frac{\alpha -1}{\tau -1}=\frac{1}{\sigma \upsilon z}$ is further verified. We further examine the shape collapse property of avalanches with sufficiently long durations using the script in the NCC toolbox [58].

## 3. Results

We study a recurrent E–I neural network whose input consists of a background part *r*_0_ and an external stimulus part *r*_1_ (Fig 1A). The network dynamics are modeled as conductance-based IF neurons with synaptic kinetics (Eqs 1 and 2). For a sensory circuit, the background input *r*_0_ can be considered as subcortical inputs from the thalamus and top-down inputs from higher cortices, so that the circuit can have spontaneous with multilevel complex dynamics. The extra stimulus *r*_1_ serves as a sensory stimulus input of the network for further processing. We study how the spontaneous and stimulus-evoked dynamics interact in this neural circuit with biologically plausible dynamic properties and particularly how stimulus-evoked dynamics depend on the circuit spontaneous properties.

We first examine the network model dynamic mode/state. These states can be best observed by studying the spontaneous dynamics with a fixed deterministic input *GO*_*i*_(*t*) = *r*_0_. Generally, the network can display four types of dynamic modes:

- *Asynchronous (AS)*: The AS state (Fig 1B) is the dynamic region of the classical E–I balanced theory [41,59], and it is characterized by the following: (i) irregular firing of neurons, in which the CV of the ISIs is close to 1.0, similar to Poisson point processes [5]; (ii) low pairwise correlation between neurons (i.e., AS); (iii) almost steady LFP; (iv) fast decay of the population activity AC (a chaotic signature); (v) exponential-like avalanche distribution (Fig 1D).
- *Sparsely synchronous (SS) state*: In the SS state (Fig 1E), also called a synchronous irregular state in some previous literature [60], neurons exhibit clustered spiking, which induces oscillatory LFPs. Because neurons in the network sparsely participate in the clustered spiking (synchrony), most of the neurons can spike irregularly. The pairwise correlation between neurons is moderate. The population activity AC features positive peaks, indicating typical temporal scales in population oscillation, which induce bimodal avalanche distribution (Fig 1D).
- *Critical (Cri) state*: The Cri state (Fig 1C) is the transition state between AS and SS states and thus possesses characteristics between both. The avalanche distribution closely follows the power law (i.e., a scale-free-like behavior, Fig 1D). The criticality properties of avalanches in the Cri state are later examined in more detail. In principle, the critical state in our model is a phenomenological concept, as the criticality in terms of statistical physics may not be well-defined in our biological neural network model. However, it was shown in our previous work [39] and will be confirmed below that such a critical synchronous transition can correspond to the Hopf bifurcation in the macroscopic field, and the latter is a true (dynamical) criticality in a nonlinear dynamical system. Thus, it is justifiable to denote such a dynamic state the critical state.
- *Periodic (P) state* (Fig 1F). This occurs when the inhibitory effect is sufficiently slow (i.e. $\tau_{d}^{I}$ is sufficiently large) in a finite-size system that an all-spike event (wherein all neurons spike in a very short time window) can trigger another all-spike event. Then, such all-spike events can occur periodically. In this case, neurons spike regularly (mostly with a regular periodicity) with very strong pairwise correlation, and the LFP also fluctuates strongly and periodically. In this state, the available avalanche size and duration can only take a few specific values.

For a fixed background input level *r*_0_, the characteristics of the dynamic mode mainly depend on the inhibition synaptic parameter $\tau_{d}^{I}$. In general, slower synaptic inhibition (increasing $\tau_{d}^{I}$) favors stronger synchrony—the degree of synchrony degree increases from the AS mode, through the Cri mode, to the SS mode and becomes strongest in the P mode. It has been shown that these dynamical states display the feature of E–I balance [39], that is, the excitatory currents received by neurons can be canceled on average by the subsequent inhibitory currents. Furthermore, as the degree of synchrony increases, the cancelation of inhibitory currents becomes slower and the E–I balance becomes “looser,” i.e., it takes longer to be restored.

The dynamics of IF oscillators in the E–I balanced region are chaotic [61], indicated by the fast decay of the population activity AC, which is a characteristic of the AS state in our network. With increasing $\tau_{d}^{I}$, the oscillation component appears and is superposed on chaotic fluctuations, and the network appears to undergo a transition from the chaotic to the periodic state. Moreover, according to the avalanche distribution properties (Fig 1D), the AS state can be called subcritical, while the SS and P states can be called supercritical.

Furthermore, we confirm the existence of the above four dynamic states in larger networks (*N* = 5000, 10,000, 15,000) (S3 Fig). Thus, these states are robust features of the model, and they are not subjected to the artifacts of small network size.

The critical value of $\tau_{d}^{I}$ that allows the Cri state can depend on the background input level *r*_0_. However, as will be shown later, the dependence on *r*_0_ for dynamic mode transitions is much less sensitive than the dependence on $\tau_{d}^{I}$; thus, we can explore the subtle difference between spontaneous and evoked dynamics by maintaining the same dynamic modes near criticality. Furthermore, even for fixed parameters *r*_0_, $\tau_{d}^{I}$, the network can still enter different dynamic modes, depending on the initial conditions. These possibilities are further explored in S1 Appendix global dynamic structure.

The above discussion concerns the dynamics under deterministic input. For networks with noisy input, the AS, Cri, and SS states can be defined by their corresponding properties. However, there is no absolute periodic state, because noise can induce occasional neuronal spikes and phase drifts of population oscillation. Moreover, noise can smoothen the transition, broadening the range of the Cri state.

### 3.1 Stimulus-induced reduction of trial-to-trial variability at criticality

We first study the TTV of the network activities, focusing on its dependence on the spontaneous dynamics and its modulation by extra stimuli. Here, TTV is defined by the intrinsic variability of the network dynamics. It is studied using numerical simulations, with different initial conditions but the same network architecture and deterministic input across trials (see Methods). We detect the TTV in the LFP by computing the cross-trial variance and the TTV in neuron spiking by cross-trial FF (see Methods). Interestingly, depending on the spontaneous dynamic mode determined by $\tau_{d}^{I}$, the network exhibits different TTV characteristics, and the TTV is further differentially modulated by external stimulus.

At the subcritical spontaneous dynamic region, the neuron spiking exhibits considerable TTV owing to the chaos in the E–I balanced region [61], whereas the TTV of the LFP is small (Fig 2A). However, the stimulus does not markedly alter the TTV of the LFP but increases that of the spikes (Fig 2D). At the supercritical spontaneous dynamic region with periodic modes, TTV still occurs because the time required for entering the periodic attractor state varies with the initial conditions (i.e., phase spread, Fig 2C and 2F). Extra stimuli enforce the network into periodic spiking with a shorter period, which increases the TTV of the spiking but reduces that of the LFP (Fig 2C and 2F). This occurrence is partially due to the phase resetting effect of the stimulus, manifested by the oscillation in the trial-averaged LFP after the stimulus onset (Fig 2C).

The critical spontaneous dynamic region around the oscillation transition is the most interesting region. Here, both spontaneous LFP and neuron spiking exhibit relatively high TTVs. Interestingly, the variance of the LFP and FF of spiking can be largely suppressed by extra stimuli (Fig 2B and 2E). Although stimuli generally increase the mean firing rate of the network, this increase is not sufficient to reduce the FF, because the variance of spiking activity increases with the mean spike count (a general property of random point processes). The TTVs of the LFP and spiking have slightly different properties. When the network is around this critical region, it exhibits Cri modes both before and after stimulus onset in most trials (Fig 2E). However, in a small proportion of trials, the network enters P modes before the stimulus onset, and extra stimuli revert the network to the Cri mode (S4A Fig). If we only consider the trials maintaining the Cri modes before the stimulus onset, the TTV of the LFP can still be reduced by stimuli, whereas the TTV of spiking cannot (S4B and S4C Fig). As will be shown later, the reduction in the LFP TTV can be understood by the noise suppression property of the extra stimulus, which is predicted by the mean-field theory (Section 3.3), and the reduction in the spiking FF depends on the global attractor dynamic structure (S1 Appendix). Moreover, shifts between dynamic modes do not occur after the stimulus onset in the subcritical and supercritical regions. The TTV at the post-stimulus critical states can still be higher than that at the subcritical states. Thus, critical networks subjected to stimuli still possess sufficient variability and flexibility for better functioning. The TTV studied here is the intrinsic variability of the network under a deterministic input. For a network subjected to a noisy Poisson input, the input variability accounts for a part of the TTV of the network dynamics. In this case, extra stimuli cannot suppress the TTV, even when the network is around the critical state (S4D and S4E Fig), primarily because the variability of the input also increases with the input strength (a property of Poisson noise).

Overall, only at the critical state can the network maintain a high internal TTV, and the TTV can be effectively suppressed by stimuli, consistent with the widely observed highly variable spontaneous states [2,62] with stimulus-suppressed TTV [17,18].

### 3.2 Stimulus modulates oscillatory frequency and preserves criticality

Neural circuits can display crackling noise activities. These activity patterns are characterized by avalanches with power-law distributions of size and duration and can be explained by critical branching theory [9]. Later experimental evidences tend to support that crackling noise activities occur in moderate synchrony regions [13,14] and obey stricter criteria predicted by criticality theory [63], such as diverse critical exponents, scaling relation, and shape collapse relation of the avalanches beyond the power-law distributions. Moreover, the critical properties occur at spontaneous states but can also be preserved after an additional stimulus onset [31,32].

Neural oscillation is another commonly observed neural dynamic activity. It is important for efficient information communication [64,65] in sensory neural circuits, and its frequency can be modulated by top-down or bottom-up inputs. For example, the gamma oscillation (defined as that with a peak frequency of 25–80 Hz) in the V1 cortex of awake-behaving macaques is stimulus-dependent [23] such that changes in stimulus contrast over time lead to reliable gamma frequency modulations on a fast time scale, suggesting that the gamma rhythm arises from local interactions between excitation and inhibition.

Although neural avalanches and neural oscillation are usually observed and studied in different experiments, they may share the same neural substrates. Our model reconciles the preservation of critical avalanches under different input strengths with the existence of network oscillations of flexibly tunable frequency. Because noise can smoothen the critical transition, a network under noisy inputs has a broader parameter range supporting Cri modes. Through numerical tests, we find that for a background input strength *r*_0_ = 0.3/*ms*, the Cri modes can be maintained when $\tau_{d}^{I}$ is 8–10.5 ms. Below, we explore the dynamic properties in this region in detail.

First, in the presence of stimuli with different strengths, circuits with spontaneous critical dynamics ($\tau_{d}^{I}=9\;ms$) first go through transient periods where larger avalanches emerge because of the strong extra input received by the network during the transient periods (see Eq (4)). Such large avalanches cause bimodal avalanche distributions (as shown in Fig 3B and in [31]). However, the network can preserve the loose E–I balance, a feature of E–I balanced networks at criticality, even during the transient period (S5 Fig). The network can re-enter criticality in the post-stimulus states after the transient period when the inputs drop to *r*_0_+*r*_1_. The post-stimulus states still feature a power–law–like avalanche size and duration distributions: *P*(*S*)~*S*^−*τ*^, *P*(*T*)~*T*^−*α*^ and 〈*S*〉(*T*)~*T*^*1*/*συz*^. To assess the power-law property, we apply the NCC toolbox [58] to find the power-law distribution ranges for avalanches obtained in the post-stimulus period under stimulus strength *r*_1_ = 0.35/*ms* (~8.5×10^4^ avalanches are used in the estimation). The toolbox provides a doubly truncated algorithm based on the maximum likelihood estimation to find the largest range that passes the truncation-based K–S statistics test [58] with *p*>0.1, meaning the data can produce a K–S statistic value that is less than the values generated by at least 10% of the power-law models in the truncated range. We find that *τ* = 1.95, *α* = 2.15, $\frac{1}{\sigma \upsilon z}=1.3$ (Fig 3B), and the scaling relation $\frac{\alpha -1}{\tau -1}=\frac{1}{\sigma \upsilon z}$ holds, with error < 0.1. The truncation ranges in this estimation are 4–49 for avalanche size and 3–42 for avalanche duration. The critical exponents of the model are more comprehensively explored in the next section. Further analysis (S6 Fig) shows that the shapes of avalanches at critical states can be collapsed into scaling functions *F* through the relation $s(t,T)=T^{\frac{1}{\sigma \upsilon z}-1}F(t/T)$, where *s*(*t*,*T*) is the time course of an avalanche with duration *T*, which substantiates the evidence of criticality. In the literature, the preservation of criticality with different input strengths is usually explained by adaptation effects such as short-term depression [31,32], which regulate the circuit to a new stable state that can defy the elevated input. Our results show that synaptic adaptation may not be necessary, as the critical region can be broad, depending on the input strength.

Furthermore, critical dynamic manifests itself as gamma network oscillation, and extra input tunes the critical circuit into another critical state with higher oscillatory frequency, depending on the input strength. We apply three levels of stimulus strength (*r*_1_ = 0.1, 0.35, 0.6/*ms*) to denote the usage of increasing contrasts (for 25%, 50%, 100%) in a macaque experiment [23]. Here, the form of the input is $r_{1}+r_{1}te^{-t/\tau_{0}}$ for *t*∈(*t*_*onset*_, *t*_*end*_). The input is first increased sharply and then steadily, to induce the ERP effect (see Methods). Fig 3C to 3H show the stimulus-induced modulation of gamma oscillation under critical dynamics with $\tau_{d}^{I}=10\;ms$. The ERP height, representing elevated post-stimulus potential and population firing rate, increases with the stimulus strength (Fig 3C and 3D). The stimulus shifts the gamma oscillation to a higher frequency, which increases with stimulus strength, as demonstrated in the power spectral density (PSD) curve (Fig 3E). This frequency modulation can be completed within 100 ms after the transient effect, and the gamma frequency immediately returns to the spontaneous level when the input is re-tuned to the background level (Fig 3F to 3H). All of these features agree with the experimental results (see Fig 1 in [23]). The stimulus-induced modulation of gamma power is most effective only at critical states. Subcritical spontaneous dynamics with the AS mode (S7A and S7B Fig) do not exhibit gamma oscillation or the modulation effect. In contrast, supercritical spontaneous dynamics (S7C and S7D Fig) show strong gamma oscillations. However, without the dynamic sensitivity at criticality, the post-stimulus frequency is not sensitively modulated by extra inputs (see the comparison in Fig 3E inset).

In summary, at the critical state, the network exhibits population oscillation with dynamic sensitivity, so that its frequency can be effectively modulated by external stimulus, while criticality is preserved.

### 3.4 Further exploration of critical avalanche dynamics

We have shown that an E–I balanced network around the critical synchronous transition region can exhibit scale-free-like avalanche dynamics. This phenomenon is further confirmed in the spontaneous critical dynamics (with $\tau_{d}^{I}=9\;ms$) for larger networks. Specifically, we use the firing rate *r*_*in*_ = 0.9/*ms* in the network with *N* = 2500 as a baseline (which is the highest input rate received by the network in the post-stimulus periods in the simulations in Fig 3), and we simulate networks with *N* = 5000, 10,000, 15,000 by keeping the scale condition *r*_*in*_~*O*(*N*) for E–I balanced networks [41] so that their inputs are set as *r*_*in*_ = 1.8, 3.6, 5.4/*ms*, respectively. Fig 4 shows that scale-free-like avalanches such that *P*(*S*)~*S*^−*τ*^, *P*(*T*)~*T*^−*α*^, and 〈*S*〉(*T*)~*T*^*1*/*συz*^ can be detected in models with network sizes *N* = 2500, 5000, 10,000 and 15,000, where avalanches are measured by adapted time bins Δ*t* = *T*_*m*_ = 0.11, 0.03, 0.02, 0.013 *ms* respectively. We apply the algorithm in the NCC toolbox [58] to find the power-law distribution ranges for networks with different sizes. The numbers of avalanches used in the estimation are approximately 6.1×10^4^, 1.2×10^5^, 7×10^5^, and 1.5×10^6^ for network sizes *N* = 2500, 5000, 10,000 and 15,000, respectively (Fig 4). The truncated ranges for estimating the critical exponents in the networks are approximately 1.5 decades for avalanche size (the truncated ranges are 5–238, 8–168, 8–268, and 13–295 for networks with *N* = 2500, 5000, 10,000 and 15,000, respectively) and one decade for avalanche duration (the truncated ranges are 4–48, 6–60, 6–62, and 9–82, respectively). The size distribution exponent *τ* is 2–3, the duration distribution exponent *α* is 2–3.5, but in all of the cases, the exponent 1/*συz*≈1.3. Interestingly, although the critical exponents are diverse, the scaling relation $\frac{\alpha -1}{\tau -1}=\frac{1}{\sigma \upsilon z}$ holds in all of the cases, with error < 0.1. Although a larger network has a considerably denser merged spike train, under the partition of a smaller time bin, the avalanche size and duration do not grow substantially larger. However, when the avalanches are measured with a fixed bin length Δ*t* = 0.02*ms*, the power-law cutoff grows as the network size increases, as shown in Fig 5A. Here, the numbers of avalanches used in the estimation are approximately 1.2×10^5^, 1.5×10^5^, 1.7×10^5^, and 1.8×10^7^, and the size exponents *τ*≈4.15, 3.7, 2.7, 2.4 (with truncated ranges 6–60, 6–90, 7–260, 7–331), duration exponent *α*≈4.65, 4.25, 3.15, 2.75 (with truncated ranges 4–27, 5–38, 5–57, 5–80), and the third exponent 1/*συz*≈1.2, 1.2, 1.3, 1.3 for network sizes *N* = 2500, 5000, 10,000 and 15,000, respectively (Fig 5A). Finally, the power-law cutoff can also grow by measuring avalanches using bins with increasing lengths. As shown in Fig 5B, when the avalanches in network with size *N* = 15,000 are measured using time bin Δ*t* = 0.4*T*_*m*_, 0.8*T*_*m*_ and 1.2*T*_*m*_, scale-free-like behavior is still maintained. Here, the numbers of avalanches used in the estimation are approximately 4.3×10^5^, 3×10^5^, and 2.2×10^5^, and the size exponents *τ*≈4.15, 3.2, 2.65 (with truncated ranges 8–80, 12–219, 9–288), duration exponent *α*≈4.95, 3.75, 3.1 (with truncated ranges 6–29, 7–72, 6–78), and the third exponent 1/*συz*≈1.2, 1.25, 1.3. Again, although the critical exponents are different in the measurements using different time bins in Fig 5, the scaling relation $\frac{\alpha -1}{\tau -1}=\frac{1}{\sigma \upsilon z}$ holds in all of the cases, with error < 0.1.

The estimated critical exponents in our model do not agree with the classical critical branching processes where *τ* = 1.5, *α* = 2, $\frac{1}{\sigma \upsilon z}=2$. The origin of the larger-than-usual critical exponents in our model is still unclear. Such large critical exponents seem to be features of critical E–I balanced networks [39]. Diverse exponents that are larger than the usual critical branching exponents and satisfy the scale relation have also been found in previous experimental data [13,31,39] and models [66] of neural avalanches.

### 3.5 Mean-field prediction of multilevel response features of E–I circuits

We have shown that an E–I circuit at (and only at) the critical oscillation transition state can simultaneously display multiple features in spontaneous and evoked states: high internal variability and stimulus-induced reduction of TTV in both the LFP and neuron spiking, effective modulation of oscillation frequency, and preservation of critical properties in the presence of a stimulus (Table 1).

**Table 1:** Comparison of dynamic features at different dynamic states.

States	Subcritical	Critical	Supercritical
Internal variability of the local field potential (LFP)	small	medium	large
Internal variability of neuron spike	medium	large	small
Stimulus-induced modulation of the trial-to-trial variability (TTV) of the LFP	no substantial change	reduce	reduce
Stimulus-induced modulation of the TTV of spiking	increase	decrease	increase
Network oscillation	no	yes	yes
Stimulus-induced modulation of oscillatory frequency	no	yes	not effectively

**Fig 6:**
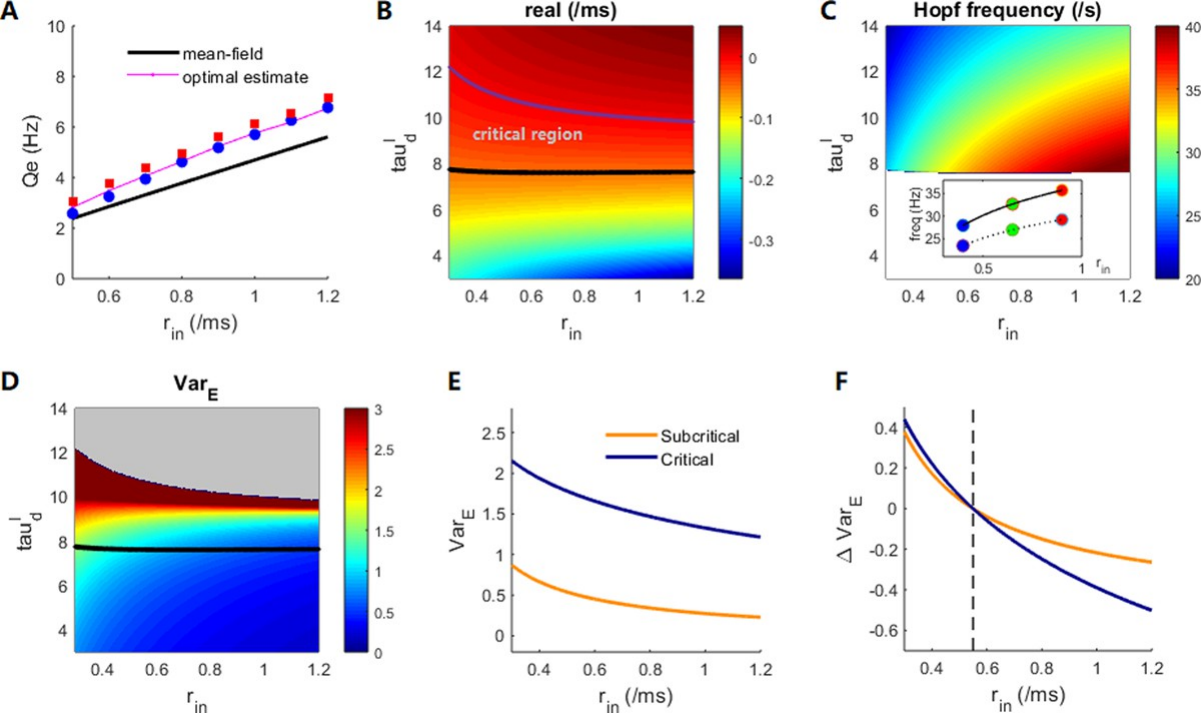
Mean-field theory prediction of the response dynamics: **(A)** Excitatory firing rates vs. input strength at the AS state, with $\tau_{d}^{I}=5\;ms$. The red/blue markers represent network simulation results under noisy/constant inputs. The black curve represents the field model fixed-point estimation under fixed *σ*_*α*_ parameters indicated at the end of this caption. The purple curve represents the result under *σ*_*α*_ parameters estimated with different *r*_*in*_ values. **(B)** The real part of the eigenvalue evaluated at equilibrium. The purple curve indicates the zero value (i.e., the deterministic Hopf bifurcation points), and the black curve corresponds to the case where the real part of the eigenvalue is −0.05. **(C)** The prediction of the population oscillation frequency *f* = *ω*/2*π*, where ω is the imaginary part of the eigenvalue at equilibrium. Only results above the effective critical black line in **(B)** are shown. The inset shows the frequencies for input strengths *r*_*in*_ = 0.4, 0.65, 0.9/*ms* (solid and dotted lines correspond to the critical and supercritical cases with $\tau_{d}^{I}=9,\;13\;ms$), similar to the inset of Fig 3E. **(D)** The linear noise prediction of *Var*(*V*_*E*_) for different $\tau_{d}^{I}$ and *r*_*in*_ values (before the Hopf bifurcation line). The black curve is the critical line in **(B)**. **(E)** Plot of *Var*(*V*_*E*_) vs. input strength *r*_*in*_ for subcritical and critical states with $\tau_{d}^{I}=4,\;9\;ms$. **(F)** Same as **(E)** but for quantity $\Delta {Var(V}_{E})={Var(V}_{E})-{{Var(V}_{E})|}_{r_{in}=0.55}$. The field equation parameters are *σ*_*e*_ = 3.2, *σ*_*i*_ = 3.8, *β* = 0.2.

To reveal the dynamical mechanism behind these multilevel features, we adopt a novel semi-analytical mean-field theory [39] to derive the macroscopic field equations (i.e., Eq (8)) corresponding to the network subjected to a fixed input strength *r*_*in*_ (see Methods). The theory relies on constructing the voltage-dependent mean firing rate, i.e., Eq (6) with presumed parameters *σ*_*E*_, *σ*_*I*_. These parameters can be optimally evaluated as $\sigma_{\alpha }=\frac{V_{th}-V_{\alpha }^{ss}}{ln[{\left(Q_{\alpha }^{ss}\right)}^{-1}-1]}\frac{\pi }{\sqrt{3}}$ [39] through numerical network simulation under AS dynamics ($\tau_{d}^{I}=5\;ms$) to obtain the mean membrane potential $V_{\alpha }^{ss}$ and firing rate $Q_{\alpha }^{ss}$. Note that such optimal estimation relies on the input strength *r*_*in*_ and the optimally constructed field equations can thus predict the network firing rate precisely (Fig 6A). Here, the prediction by the field equation is based on its fixed-point values. However, the firing rate (Fig 6A), and especially, its linear relation to the input strength *r*_*in*_, can satisfactorily be predicted using other fixed *σ*_*E*_, *σ*_*I*_ values.

The field equation provides a method to analyze the stability of the equilibrium obtained by solving Eq (9) by checking the eigenvalue of the Jacobian matrix (Eq (10)). The transition from AS to oscillatory modes corresponds to the Hopf bifurcation in the field equations [39], indicated by a pair of dominant complex eigenvalues *α*±*iω* crossing the imaginary axis. The Hopf bifurcation indicates that the stable fixed point of the field equations will give way to a stable periodic solution, whose amplitude grows from zero. Furthermore, the frequency of the periodic motion can be estimated as *ω*/2*π* in the linear order, and we term *f* = *ω*/2*π* the Hopf frequency. As depicted in Fig 6B, the Hopf stability (the real part *α* of the eigenvalue) is mainly governed by $\tau_{d}^{I}$, while its dependence on the input strength *r*_*in*_ is much weaker. Because of the noise perturbation, the criticality property does not emerge at the Hopf bifurcation point but before the bifurcation. If we heuristically assume a suitable threshold of the real part of the eigenvalue, e.g., −0.05, then the field model with an eigenvalue real part larger than this threshold can be assumed to be driven by noise to exhibit critical properties. Then, we can define the critical region by a critical line above which the system enters criticality (Fig 6B). For certain $\tau_{d}^{I}$ values, this critical region spans a broad range with respect to the input strength, which explains the criticality preservation of the neural network for increased input strengths (Fig 3B). However, the Hopf frequency ($f=\frac{\omega }{2\pi }$), which lies in the gamma range around critical states, increases with the input strength, and it is more sensitive for smaller $\tau_{d}^{I}$ values (Fig 6C). This explains how stimuli increase the post-stimulus gamma frequency most effectively at the critical point (Fig 3E to 3H).

Before the Hopf bifurcation, the derived deterministic field equations do not exhibit TTV because its dynamic converges to the fixed point in a short time. However, the GWN can induce TTV in the field equations (see Methods). Due to ergodicity, the cross-trial fluctuation of the LFP at a specific time is equivalent to the single-trial fluctuation across time. Thus, in the presence of noise in the field equations, the magnitude of the dynamic fluctuation of *V*_*E*_ is equivalent to the TTV of the LFP in the microscopic neural network; this fluctuation is indicated by the LNA of the activity fluctuation around the equilibrium (see Methods). The steady-state fluctuation variance *Var*(*V*_*E*_) can be obtained by solving the Lyapunov equation Eq (13) under different $\tau_{d}^{I}$ and *r*_*in*_ (Fig 6D). The *Var*(*V*_*E*_) increases with $\tau_{d}^{I}$ before the Hopf bifurcation, because the fluctuation is larger when the equilibrium stability is weaker (closer to bifurcation). For the same $\tau_{d}^{I}$, the fluctuation variance *Var*(*V*_*E*_) appears smaller for larger inputs. The increase in the input strength can reduce the dynamic fluctuation and thus the TTV, which represents the noise reduction mechanism. The LNA shows the following properties: 1) Under the same input strength, *Var*(*V*_*E*_) is larger at the critical state (closer to the Hopf bifurcation) than at the subcritical state (far below the Hopf bifurcation) (Fig 6E). 2) The fluctuation suppression given by $\Delta {Var(V}_{E})={Var(V}_{E})-{{Var(V}_{E})|}_{r_{in}=0.55}$ (where *r*_*in*_ = *r*_0_ = 0.55/*ms* is the spontaneous input level used in Fig 2) is larger at the critical state than at the subcritical state (Fig 6F). This explains why E–I networks poised at criticality have both high internal variability and strong stimulus-induced suppression of variability in the LFP (Fig 2B and 2E).

However, the macroscopic field equations here cannot directly reflect the properties of spikes in the E–I network. In the network, high magnitudes in the LFP correspond to the clustered spiking of neurons (Fig 1), so that the spike property can be partially understood from the LFP property. Nevertheless, the TTV of neuron spiking, measured by FF, is more intricate, and the global dynamic structure in S1 Appendix explains the stimulus-induced reduction of FF.

Finally, the quantities of fluctuation and the stimulus-induced reduction of fluctuations are determined by the imposed noise strength *β* in the field equations, while the qualitative conclusion that fluctuation can be most suppressed around critical dynamic states does not depend on *β* (see S8 Fig). The sensitivity of the bifurcation point on the effective parameters *σ*_*E*_, *σ*_*I*_ for constructing the field equations is further examined (shown in S9 Fig).

In summary, through the mean-field theory, a map from the E–I spiking neural network to neural field equations can be effectively constructed. The network properties in spontaneous and evoked states—namely LFP variability, oscillatory frequency, and criticality property—can be predicted under different background dynamic modes determined by $\tau_{d}^{I}$.

## 4. Discussion

In this paper, we study the stimulus–response relationship in different spontaneous states of E–I balanced neural networks. It is found that E–I circuits poised around critical states can reproduce multilevel dynamic features, namely neuron spiking, firing rate, neural avalanches, LFP, and network oscillation frequency, etc., in accordance with different experimental observations. These multiple facets are unifiedly predicted by a novel mean-field theory. Below, we compare our model and theory with those of previous studies.

### 4.1 Neural oscillation and criticality

Cortical neural networks exhibit cognition-related rhythmic activities in terms of population oscillation [33]. Neural oscillations within the gamma frequency range are of particular interest as they are found to associate with high-level cognitive functions such as attention [67], memory [68,69], and perception [70]. Gamma oscillation can arise from the E–I interaction in the local E–I neural circuit [40,71] with sparsely synchronous but irregular spiking of neurons [72]. The dynamic mechanism by which the balance of excitatory and inhibitory currents in the circuit induces spike irregularity has been theoretically established as the classical E–I balance theory [41,59]. The irregular neuronal spiking can also organize as scale-free avalanches [57]. Traditionally, critical avalanches are explained by critical branching process theory [9]. Other studies have shown that critical oscillation transition theory [39,66,73] can better account for the critical phenomena. Under this scenario, the scale-free-like avalanches of the neurons and scale-dependent gamma oscillations of the network can coexist at the critical synchronous transition states of E–I balanced neural networks [39,74]. In this paper, we find that in this biologically plausible dynamic critical region of the spontaneous state, a network in the presence of extra inputs can self-organize into critical states characterized by different gamma frequencies.

In the traditional theory of criticality in neural systems [75], the critical state is often confined to a small parameter range, which converges to a critical point as the system size increases. It arises a question about how neural systems exposed to diverse environments can achieve criticality. An idea is the self-organized criticality theory [76], which holds that critical states are stable attractors for different external conditions. A common way to maintain the critical state in neural networks in the presence of perturbation is through synaptic plasticity, as demonstrated in previous models [31,77,78]. In these models, networks under various initial conditions can evolve from non-critical transient states to a critical stationary state, determined by the plasticity effect. In contrast, our model shows that E–I networks intrinsically adapt to different input strengths, allowing a critical region with different dynamic details (e.g., network oscillation frequency). Thus, the findings here indicate that the maintenance of criticality in E–I balanced neural networks does not necessarily require (explicit) adaptation mechanisms. Another approach for preserving criticality for broad parameter ranges without an adaptation mechanism is the adoption of Griffiths phases [79], in which criticality extends from a singular point to a stretched region in networks with hierarchical/modular structures.

The critical state has been proposed to have advantages in information processing optimum in dynamical range [28,29,32], information capacity and transmission [80], complexity [81], and information representation [38,82]. However, previous studies have mostly focused on the variability aspect of criticality. Neural systems require not only flexibility but also reliability to process input signals. Our work here further reveals the advantage of criticality in terms of dynamic reliability in response to stimuli. First, the complexity of the spontaneous critical state implies a certain level of TTV, while a stimulus can reduce the TTV and thus enhance reliability in the evoked state, which is ideal for information processing. Second, the critical states in our E–I network exhibit population oscillations whose frequency is sensitive to external modulation. A previous study proposed that the stimulus-induced modulation of the gamma frequency of the network can be leveraged to encode the information of the stimulus [35]. Thus, this type of criticality in neural networks is also beneficial for information encoding and decoding. Indeed, it has been shown that E–I balanced networks with critical features found here (e.g., gamma oscillation and weak pairwise correlation) can achieve most efficient coding [65]. Such dynamic features are also beneficial to encode memory in networks through spike-timing-dependent plasticity [83].

### 4.2 Mechanism of stimulus-induced reduction of trial-to-trial variability

Different modeling frameworks have been proposed to explain the widely observed and behavior-relevant stimulus-induced reduction of TTV. Previous theories can be roughly classified into three classes.

The first mechanism is chaos suppression. It is known that the dynamics of randomly coupled rate units can undergo a transition from a steady state to a chaotic state as the coupling grows [84]. Later works [85,86] have shown that chaos in such networks can be suppressed when sufficiently strong oscillatory inputs are applied, thus reducing the TTV. The resonant phenomenon predicted by this theory has also been experimentally observed [87]. However, whether chaos suppression can be achieved in spiking neural networks is unclear. Spiking E–I neural networks can also show similar chaotic firing rate fluctuation induced by strong recurrent coupling [88]. However, it was shown that this chaotic firing rate fluctuation dynamic region cannot support the stimulus-induced reduction of TTV [89].

The second mechanism occurs in networks with multiple attractors. Spontaneous cortical dynamics exhibit complex patterns explained by dynamic trajectories wandering between different attractors [90]. Spiking neural networks with multiple attractors can result in high TTV at ongoing states, characterized by frequent transitions between different attractors, whereas extra stimuli can stabilize the network toward particular attractor(s) and thus reduce the TTV [89]. A typical example is neural networks with clustered topology [91,92], where extra inputs can bias the dynamics to particular cluster(s), reducing the TTV in the presence of stimulus.

The third mechanism is noise reduction, which is achieved in noisy neural field models with specific properties. In these models, the increase in input (parameters) can lead to the reduction of fluctuation in dynamic variables in the equations, which is often explainable by LNA. Typical examples include a large-scale human cortical model [93] and the stochastic stabilized supralinear network model [94]. Our derived field equations from the mean-field theory also obey this mechanism.

In our study, we observe pronounced stimulus-induced reductions of TTV in both LFP and spiking when the E–I network is poised at the critical oscillatory transition region, but the two reductions have slightly different properties. The TTV of the LFP decreases when extra stimuli drive the network to a higher frequency state. This is demonstrated by the noise reduction in the corresponding effective macroscopic mean-field equations. In contrast, the spiking TTV decreases when extra stimuli revert a small proportion of trials with periodic modes back to critical modes. In such spontaneous dynamic region, two types of attractors (one chaotic, the other periodic) exist in the network (see S1 Appendix). Thus, the reduction in the spiking FF in our model also involves the above-mentioned second mechanism. The periodic mode with eliminated chaos is induced by sufficiently long inhibition owing to the finite network size. Thus, the present finding is a counterintuitive result that TTV can be reduced by enhancing chaos. It should be noticed that the dynamic feature of our critical E–I network is similar to the spiking version of a stochastic stabilized supralinear network model in the literature [94] in terms of weak population oscillation and loose E–I balance. However, different from the presumed stochasticity for generating variability in that stochastic model, our model exhibits internal variability from the E–I balance–induced chaotic spike time. Overall, the presented critical E–I network model provides a novel TTV reduction mechanism in spiking neural networks that is related to but different from previous theories.

### 4.3 Outlook

The E–I balanced neural network model incorporating biological synapse kinetics with its effective mean-field approximation theory captures biologically realistic features in terms of dynamic behavior, variability, and criticality. It is thus a promising modeling framework to study the multilevel stimulus–response dynamics and their relationship with cognition, brain function, and brain disorders. The relationships between spontaneous brain activity and its behavioral outcomes have been widely observed in brain functional connectome [95,96].

A recent study [37] proposed a framework based on the linear Wilson–Cowan equation and fluctuation-dissipation theorem in nonequilibrium statistical physics to understand the relationship between the ongoing and evoked dynamics. From our derived field equations, one may further derive the autocorrelation and response function based on the fluctuation-dissipation theorem [37]. This may provide insights into how the stimulus–response properties can be predicted from the spontaneous dynamics [37] and would be an interesting direction to be explored in the future. On the other hand, our model exhibits unusual critical exponents, as shown in Fig 4. These exponents may also depend on the network details such as the synaptic coupling parameters, time scale of synapses, and network size. The relationship between the network dynamic properties and the value of the critical exponents is presently unclear and it should require further efforts to explore.

The model and analysis in this paper can be extended to incorporate realistic components for task processing. For example, this extension may explore the dynamics of the evoked up-states in working memory recall [97] and decision making [98] and how they interact with or depend on the dynamics of the corresponding spontaneous states. It is also interesting to extend the theory to spatially extended networks that allow the propagation of waves [99,100] related to experimentally observed cortical waves [101]; through such studies, the dimensionality [102] and shared variability [103] properties of neural dynamics during spontaneous and evoked states can be further explored. To investigate task processing dynamics between brain regions, the present model can be generalized to large-scale coupled neural fields incorporating brain connectomes [104]. Moreover, future studies can adopt the information theory approach [82,105] to examine the functional benefits at the critical states of E-I networks. The above-mentioned potential directions will be interesting to explore in the future.

## Supporting information

S1 Fig TTV property of LFP when the effect of inhibitory neurons is taken into account. (PDF)

S2 Fig Effect of the number of grouped neurons on measuring FF. (PDF)

S3 Fig The four dynamic states in networks with larger sizes. (PDF)

S4 Fig Further exploration of trial-to-trial variability under critical dynamic region. (PDF)

S5 Fig Critical states maintain the loose E-I balance during stimulus onset. (PDF)

S6 Fig Shape collapse properties of avalanches at critical state. (PDF)

S7 Fig The frequency properties under subcritical and supercritical dynamics. (PDF)

S8 Fig The dependence on noise strength of the linear noise approximation results. (PDF)

S9 Fig Dependence on the input strength of the effective parameters *σ*_*E*_, *σ*_*I*_ and the sensitivity of the critical bifurcation points. (PDF)

S1 Appendix The global dynamic structure. (PDF)
