# Supplementary material for: Criticality enhances the multilevel reliability of stimulus responses in cortical neural networks
Source: PLoS Comput Biol. 2022 Jan 31;18(1):e1009848. doi: 10.1371/journal.pcbi.1009848 (PMC8830719; doi:10.1371/journal.pcbi.1009848)
Supplement: S1 Fig — (PDF) [file pcbi.1009848.s001.pdf]

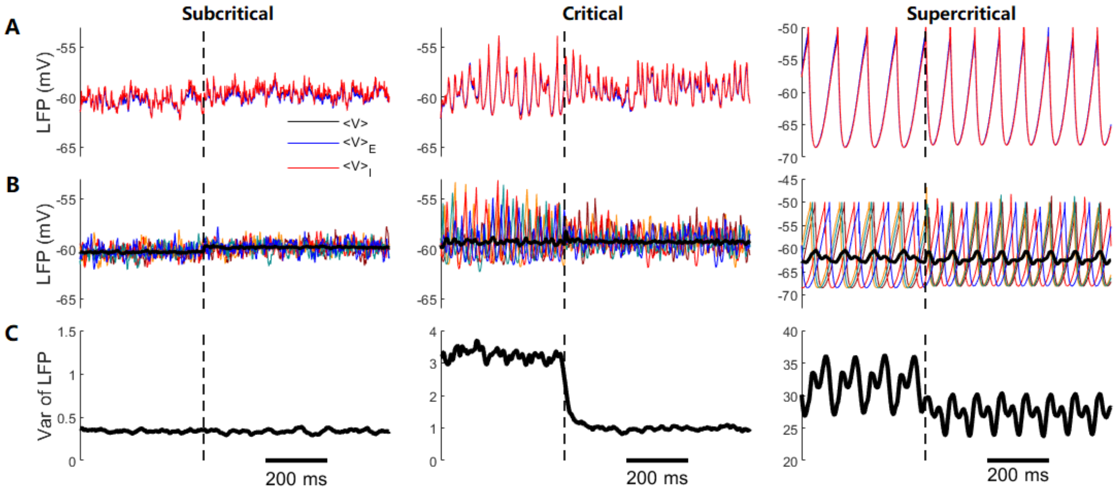

S1 Fig. TTV property of LFP when the effect of inhibitory neurons is taken into account. **(A)** Example of the single trial LFP defined by the $\langle V_i \rangle_{i \in all\, neurons}$ (black), $\langle V_i \rangle_{i \in Exc\, neurons}$ (blue) and $\langle V_i \rangle_{i \in Inh\, neurons}$ (red). The LFPs defined by these three different methods are almost identical. **(B, C)** show the same results as in Fig 2A to 2C, except that the LFP here is defined by $\langle V_i \rangle_{i \in all\, neurons}$. **(B)** the LFP of 5 single trials (labeled in different colors) and the all-trial-averaged LFP (bold black line). **(C)** the cross-trial variance of LFP. The conclusion in Fig 2 is still the same when the effect of inhibitory neurons is taken into the LFP. All parameters are the same as in Fig 2.
