# Supplementary material for: Criticality enhances the multilevel reliability of stimulus responses in cortical neural networks
Source: PLoS Comput Biol. 2022 Jan 31;18(1):e1009848. doi: 10.1371/journal.pcbi.1009848 (PMC8830719; doi:10.1371/journal.pcbi.1009848)
Supplement: S2 Fig — (PDF) [file pcbi.1009848.s002.pdf]

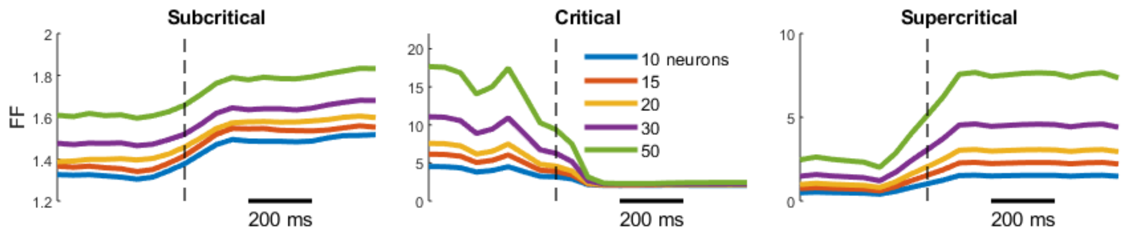

S2 Fig. Effect of the number of grouped neurons on measuring FF. The cross-trial Fano factor FF of spiking time measured by grouping different number of neurons. Parameters are the same as in Fig 2**.** Different colored curves represent the results of different numbers of neurons used (labeled in the middle subfigure). The magnitude of FF increases with the measured neuron number n whereas the stimulus-modification effect is robust with respect to n. Results in Fig 2 are with neuron number $n = 5$.
