# Supplementary material for: Criticality enhances the multilevel reliability of stimulus responses in cortical neural networks
Source: PLoS Comput Biol. 2022 Jan 31;18(1):e1009848. doi: 10.1371/journal.pcbi.1009848 (PMC8830719; doi:10.1371/journal.pcbi.1009848)
Supplement: S3 Fig — (PDF) [file pcbi.1009848.s003.pdf]

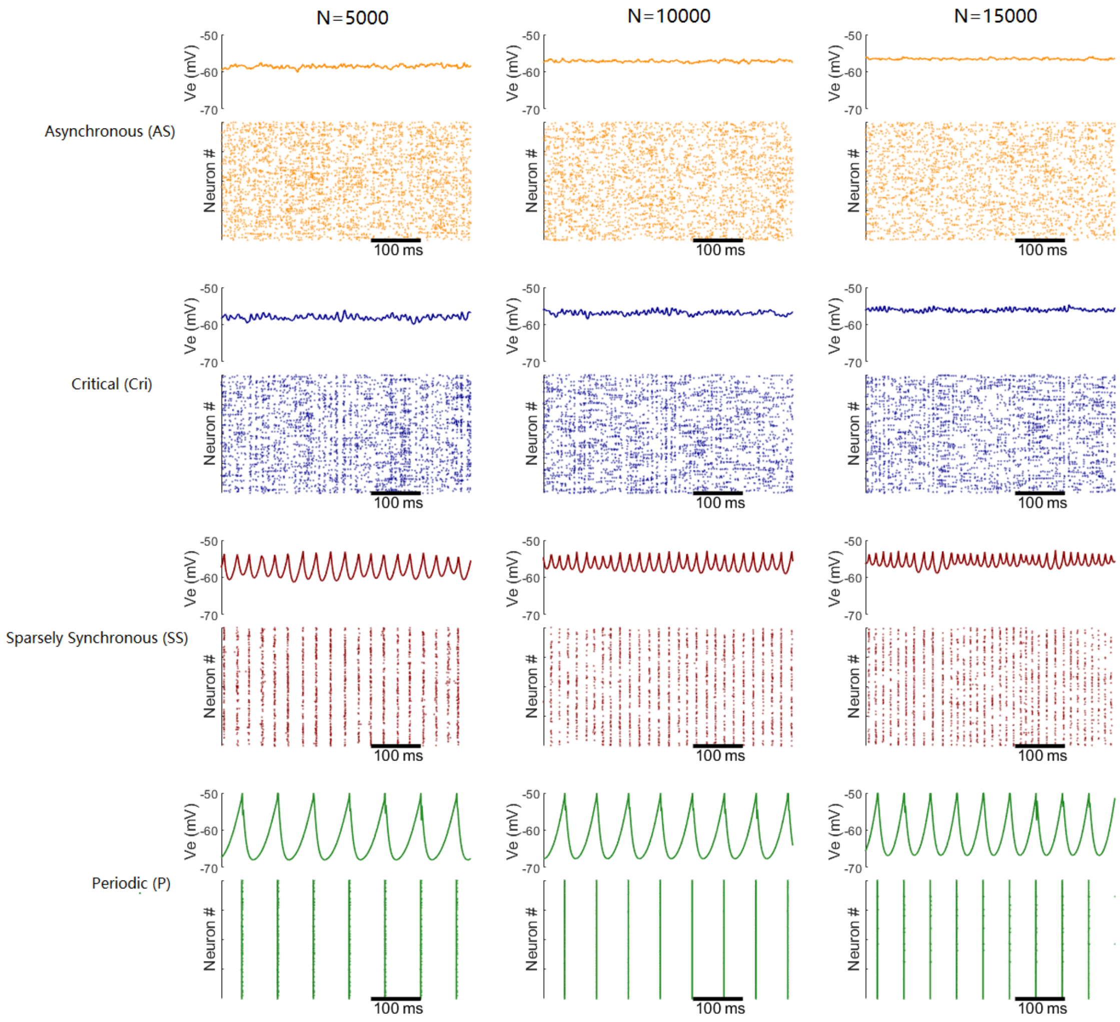

S3 Fig. The four dynamic states in networks with larger sizes. We simulated networks with larger sizes of $N = 5000, 10000, 15000$. With the increase of synaptic parameter τ_d^I, four dynamic states AS, Cri, SS and P can be identified as in Fig 1 in the main text. Thus, these states are robust feature of the model. In the raster plot of each case, only the spikes of 2000 neurons are shown for clarity. Parameters are as follows: $r_0 = 1.6\ /ms, \tau_d^I = 4, 8, 11, 14\ ms$ for $N = 5000; r_0 = 3.2\ /ms\ , \tau_d^I = 4,8, 10, 13 ms$ for $N = 10000;\ \ r_0 = 4.8\ /ms\ , \tau_d^I = 4, 8, 9.5, 11\ ms$ for $N = 15000$.
