# Supplementary material for: Criticality enhances the multilevel reliability of stimulus responses in cortical neural networks
Source: PLoS Comput Biol. 2022 Jan 31;18(1):e1009848. doi: 10.1371/journal.pcbi.1009848 (PMC8830719; doi:10.1371/journal.pcbi.1009848)
Supplement: S4 Fig — (PDF) [file pcbi.1009848.s004.pdf]

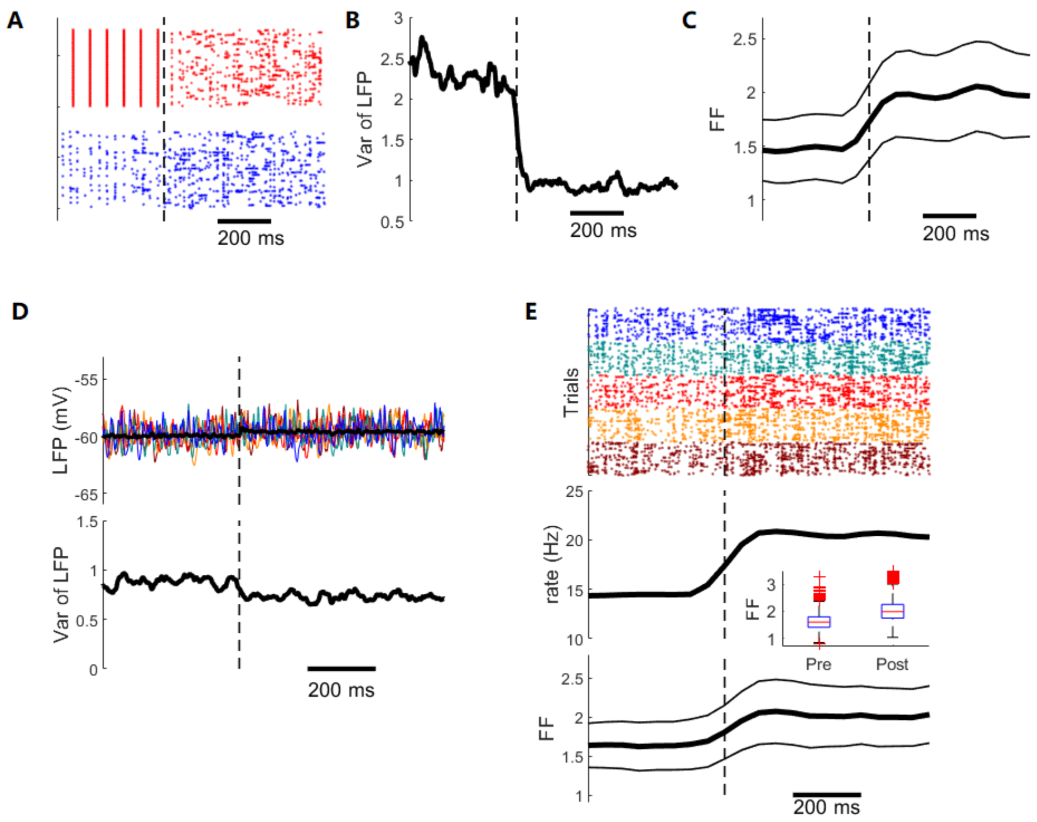

S4 Fig. Further exploration of trial-to-trial variability under critical dynamic region. All model parameters are the same as in Fig 2B and 2E. The results in **(A-C)** are from the model with deterministic input. **(A)** Examples of two trials. In the upper trial, the network enters periodic mode before stimulus onset and extra stimulus drives it back to Cri mode. The firing rate actually decreases in this case. In the lower trial, the network maintains the critical mode as before stimulus onset. **(B)** the cross-trial variance of LFP. **(C)** Fano factor FF (flanking traces are the value of $\pm$ std.). In the TTV computation in **(B, C)**, only trials maintaining critical modes before stimulus onset are considered. Under such consideration, the variance of LFP decreases after stimulus whereas the FF of spike increases. The results in **(D, E)** are from a model with noisy input. **(D)** the LFP of 5 single trials (labeled in different colors) and the all-trial-averaged LFP (bold black line), and the cross-trial variance of LFP. **(E)** the raster plots of 300 Exc neurons in 5 different trials (labeled in different colors), the trial-averaged firing rate and Fano factor FF (flanking traces are the value of $\pm$ std). When the model is subjected to noisy inputs, additional TTV source comes from the different realization of input noise in each trial. In this case, the variability of the input also increases with the input strength (the property of Poisson noise) and the TTV cannot be reduced by increasing extra stimulus.
