# Supplementary material for: Criticality enhances the multilevel reliability of stimulus responses in cortical neural networks
Source: PLoS Comput Biol. 2022 Jan 31;18(1):e1009848. doi: 10.1371/journal.pcbi.1009848 (PMC8830719; doi:10.1371/journal.pcbi.1009848)
Supplement: S5 Fig — (PDF) [file pcbi.1009848.s005.pdf]

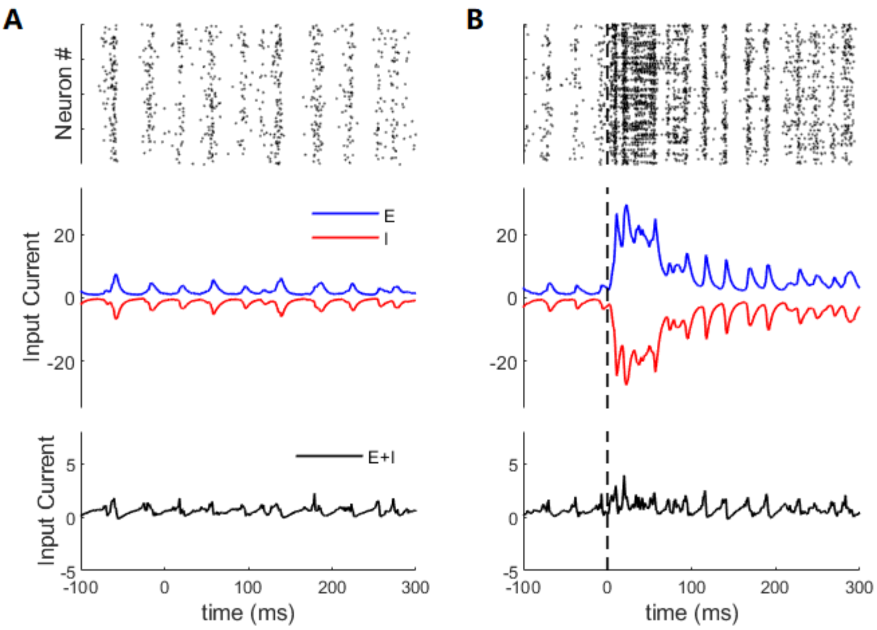

S5 Fig. Critical states maintain the loose E-I balance during stimulus onset. (A) The example of spontaneous dynamics of critical state ($\tau_d^I = 10ms$) in a network with noisy background input with strength $r_0 = 0.3/ms$. The upper panel shows the spike raster plot of Exc neurons. The middle panel shows the averaged excitatory current $\langle (V_E^{rev} - V_i)[g_{\alpha o} GO_i(t) + g_{\alpha E} GE_i(t)] \rangle_i$ and inhibitory current $\langle (V_I^{rev} - V_i) g_{\alpha I} GI_i(t) \rangle_i$ received by a neuron in the network. The lower panel shows the summation of the Exc and Inh currents. It fluctuates around zero but fluctuates more positively when there is clustered spiking in the network, suggesting a loose E-I balance state [1]. **(B)** Same plots as **(A)** when extra stimulus $r_1 = 0.35\ /ms$ is applied at $t = 0$, referring to Fig 3C. Stimulus increases the firing rates of neurons, resulting in both higher excitatory and inhibitory currents, while the loose E-I balance maintains during stimulus onset.

Reference

1. Liang J, Zhou T, Zhou C. Hopf Bifurcation in Mean Field Explains Critical Avalanches in Excitation-Inhibition Balanced Neuronal Networks: A Mechanism for Multiscale Variability. Front Syst Neurosci. 2020;14: 580011. doi:10.3389/fnsys.2020.580011
