# Supplementary material for: Criticality enhances the multilevel reliability of stimulus responses in cortical neural networks
Source: PLoS Comput Biol. 2022 Jan 31;18(1):e1009848. doi: 10.1371/journal.pcbi.1009848 (PMC8830719; doi:10.1371/journal.pcbi.1009848)
Supplement: S6 Fig — (PDF) [file pcbi.1009848.s006.pdf]

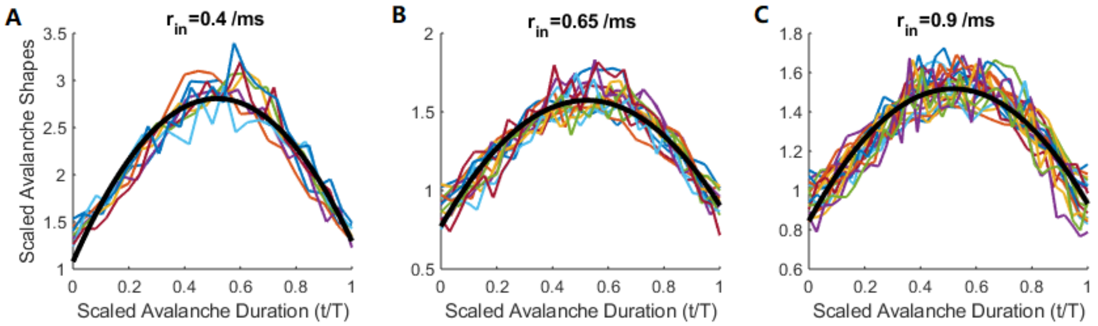

S6 Fig. Shape collapse properties of avalanches at critical state. Critical theory predicts that the time course of an avalanche with duration T, denoted as $s(t,T)$, can be collapsed into the same scale function $F(x)$ through the relation $s(t,T) = T^{\frac{1}{\sigma\nu z}-1}F(t/T)$. In order to get enough avalanches with sufficient long duration, we simulate the network with 20s with input strength $r_{in} = 0.4, 0.65, 0.9\ /ms$ (the post-stimulus input strengths used in Fig 3), $\tau_d^I = 9ms$, and combine the results of 6 trials. Only avalanches with duration T such that i) $T \geq 20$ and ii) there are at least 50 avalanches with duration T, are picked out for analysis. Results for $r_{in} = 0.4, 0.65, 0.9\ /ms$ are shown in **(A-C),** respectively. The black bold curves indicate the fitting of the universal scale functions F.
