# Supplementary material for: Criticality enhances the multilevel reliability of stimulus responses in cortical neural networks
Source: PLoS Comput Biol. 2022 Jan 31;18(1):e1009848. doi: 10.1371/journal.pcbi.1009848 (PMC8830719; doi:10.1371/journal.pcbi.1009848)
Supplement: S7 Fig — (PDF) [file pcbi.1009848.s007.pdf]

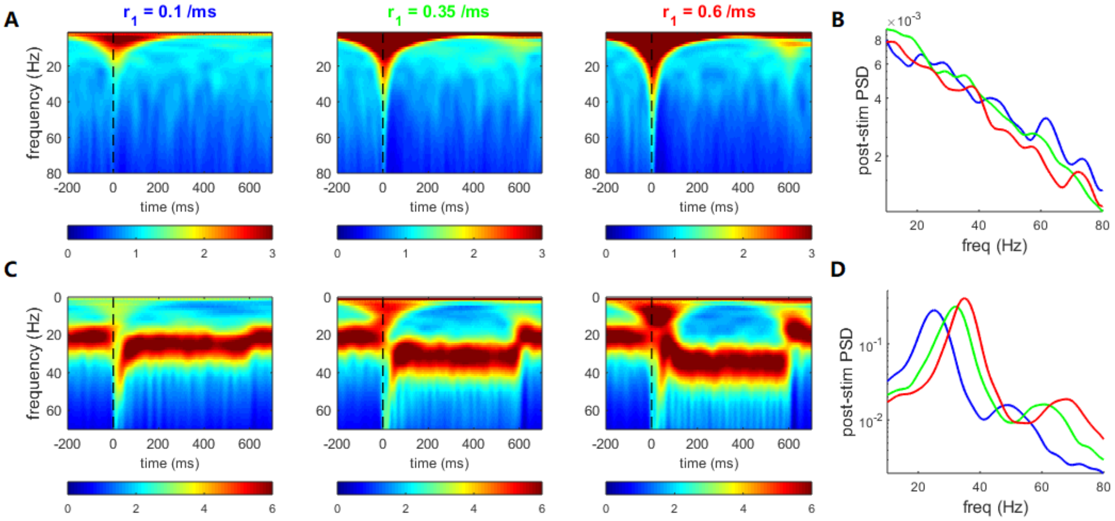

S7 Fig. The frequency properties under subcritical and supercritical dynamics. (A) The time-evolution of powers of different oscillation frequencies of LFP for different input strengths. **(B)** The PSD of post-stimulus LFP, measured in 100~600 ms after stimulus onset. Blue, green, red curves are for post-stimulus strength $r_1 = 0.1, 0.35, 0.6\ /ms$ repectively. The results of **(A, B)** are under subcritical dynamics with $\tau_d^I = 5\ ms$. Subcritical dynamics does not support gamma network oscillation nor does the stimulus modification effect. **(C, D)** Similar as **(A, B)** but for supercritical dynamics with $\tau_d^I = 13\ ms$. For supercritical dynamics, there is also stimulus modification effect of gamma power but the effect is much weaker, see comparison in the inner panel in Fig 3E. Here, background input strength is $r_0 = 0.3/ms$.
