# Supplementary material for: Criticality enhances the multilevel reliability of stimulus responses in cortical neural networks
Source: PLoS Comput Biol. 2022 Jan 31;18(1):e1009848. doi: 10.1371/journal.pcbi.1009848 (PMC8830719; doi:10.1371/journal.pcbi.1009848)
Supplement: S8 Fig — (PDF) [file pcbi.1009848.s008.pdf]

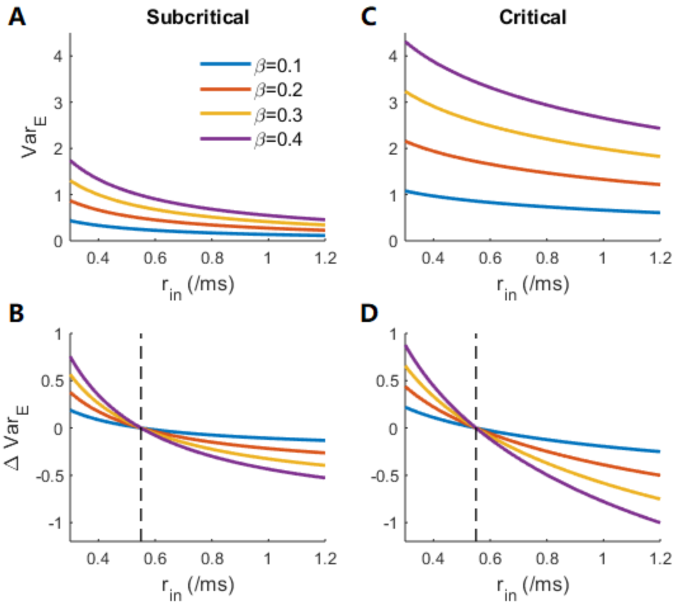

S8 Fig. The dependence on noise strength of the linear noise approximation results. (A, B) The $Var(V_E)$ and $\Delta\, Var(V_E) = Var(V_E) - Var(V_E)|_{r_{in}=0.55}$ versus input strength r_{in} for subcritical dynamic $\tau_d^I = 4\, ms$. Different colored curves are results under different noise strength β labeled in **(A)**. **(C, D)** Same as **(A, B)** but for critical dynamic with $\tau_d^I = 9\, ms$. Larger noise strength β induced larger $Var(V_E)$ and $\Delta\, Var(V_E)$, while irrespective of noise strength, the fluctuation ($Var(V_E)$) and stimulus-reduction of variability ($\Delta\, Var(V_E)$) at critical dynamics is larger than that at subcritical dynamics.
