# Supplementary material for: Criticality enhances the multilevel reliability of stimulus responses in cortical neural networks
Source: PLoS Comput Biol. 2022 Jan 31;18(1):e1009848. doi: 10.1371/journal.pcbi.1009848 (PMC8830719; doi:10.1371/journal.pcbi.1009848)
Supplement: S9 Fig — (PDF) [file pcbi.1009848.s009.pdf]

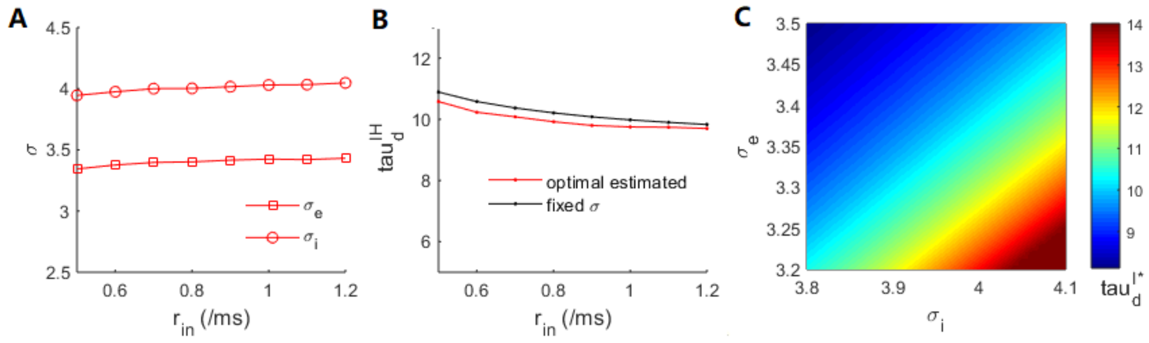


**S9 Fig. Dependence on the input strength of the effective parameters $\boldsymbol{\sigma_E, \sigma_I}$ and the sensitivity of the critical bifurcation points. (A)** Numerical estimation of $\sigma_E, \sigma_I$ with different background input strength by formula $\sigma_\alpha = \frac{V_{th} - V_\alpha^{SS}}{\ln[(Q_\alpha^{SS})^{-1} - 1]} \frac{\pi}{\sqrt{3}}$. **(B)** The Hopf bifurcation value $\tau_d^{IH}$. Red curve is the results using $\sigma_E, \sigma_I$ values in **(A)** and black curve is using fixed $\sigma_E = 3.2, \sigma_I = 3.8$. One can see that the numerically estimated $\sigma_\alpha$ values are almost the same within the background input range so that using fixed $\sigma_\alpha$ values under different input strength can also achieve good predictive quality. **(C)** Dependence on $\sigma_E, \sigma_I$ of the Hopf bifurcation value $\tau_d^{IH}$. If $\sigma_E, \sigma_I$ are taken as the free parameters, the $\tau_d^{IH}$ depends mainly on the value of $\sigma_E - \sigma_I$. Thus, suitable choices of $\sigma_E, \sigma_I$ are important for achieving good prediction quality of the field equations. Here, $r_{in} = 0.7\ /ms$ is used in **(C)**.
