# Supplementary material for: Criticality enhances the multilevel reliability of stimulus responses in cortical neural networks
Source: PLoS Comput Biol. 2022 Jan 31;18(1):e1009848. doi: 10.1371/journal.pcbi.1009848 (PMC8830719; doi:10.1371/journal.pcbi.1009848)
Supplement: S1 Appendix — (PDF) [file pcbi.1009848.s010.pdf]

S1 Appendix The global dynamic structure

In this Appendix, we explore the global dynamic structure of the spiking network. This analysis provides insights for how the TTV of neuron spiking, measured by FF, is reduced. As shown in S4 Fig and discussed previously, the spike FF can only be reduced when extra stimuli drive a small proportion of trials, which enters P modes before stimulus onset, back to Cri modes. As will be shown below, this can be understood from the change of attractor structure of the system.

Consider again the network with deterministic input $GO_i(t) = r_{in}$. We have shown that small τ_d^I favors AS mode and large τ_d^I favors SS or even P modes, whereas the dynamic modes also depend on the initial conditions. We can test the minimal value of τ_d^I required for the existence of P mode, denoted as τ_d^{I*}, as follows. For certain input strength r_{in}, the network is firstly simulated by a large τ_d^I which guarantees that the network can enter P mode quickly for any initial conditions. Then τ_d^I is tuned to a smaller value $\tau_d^{I\#}$, and we can observe whether 1) the network shifts to chaotic (AS, Cri, SS) modes, or 2) the network maintains P mode but with higher frequency, referring to Fig a (A). Then, τ_d^{I*} is the smallest $\tau_d^{I\#}$ where option 2) will set in. Next, there is another value, τ_d^{I**}, the maximal τ_d^I value that allows the existence of chaotic modes, which means that networks with τ_d^I larger than τ_d^{I**} can only maintain P mode. Finally, for the chaotic modes, there is a transition from AS to SS modes through Cri modes at another critical value τ_d^{IH}, which is interpreted by Hopf bifurcation in the mean-field.

Though the precision of this critical parameter values $\tau_d^{I*}, \tau_d^{I**}$and τ_d^{IH}suffers from numerical errors, we can detect their approximate values by simulations as follows. To determine τ_d^{I*}, we simulate the network for 3s for 30 trials. In the initial 1s, the network is first simulated by $\tau_d^I = 14ms$, then τ_d^I is changed to a smaller value $\tau_d^{I\#}$. We consider τ_d^{I*} as the minimal value of $\tau_d^{I\#}$ such that the network maintains P mode at the end of the simulation of all trials. To determine τ_d^{I**}, we again simulate the network for 3s for 30 trials for a given τ_d^I. We consider τ_d^{I**} as the maximal value of τ_d^I such that the network maintains chaotic modes at the end of the simulation of at least in one trial. Finally, we consider τ_d^{IH} as the minimal value of τ_d^I such that the chaotic mode of the network is Cri mode (note that in general there is a small range of τ_d^I that allows Cri mode in the presence of noise). Interestingly, these critical values depend on the level of background input r_{in}. The value of τ_d^{I*} , τ_d^{I**} , τ_d^{IH} from $r_{in} = 0.41/ms$ (the minimal input to launch the network activity) to $r_{in} = 1.3/ms$ is shown at Fig a (B). It can be seen that both critical values increase with input strength. Note that Cri mode (and thus τ_d^{IH}) does not exist for too weak input r_{in}.

The dependence of these critical values on the input strength provides insight for the TTV reduction around critical states in Fig 2. For relatively small background input $r_{in} = r_0 = 0.55/ms$, it is found that $\tau_d^{I*} < \tau_d^{IH} < \tau_d^{I**}$. In this case, if the synaptic decay time τ_d^I of the network is close to τ_d^{IH}, P mode attractors coexist with Cri mode attractors, referring to Fig a (C). By taking it as spontaneous state and increasing the input to $r_{in} = r_0 + r_1 = 0.75/ms$, it is found that the value of τ_d^{IH} does not change much whereas the values of $\tau_d^{I*}, \tau_d^{I**}$ increase such that $\tau_d^{IH} < \tau_d^{I*} < \tau_d^{I**}$, referring to Fig a (D). In this case, there are only Cri mode attractors around τ_d^{IH}. In general, the TTV of entering different types of attractors is much greater than that of wandering within the same attractor. We found that FF of spiking is reduced (see Fig 2E) when extra stimuli drive the network from this double-attractors (one chaotic and another periodic) region to the single, chaotic attractor region. However, the reduction of Var of LFP is not limited to this case (see Fig 2B and S2B Fig). As stimuli can increase the network oscillatory frequency and networks with higher frequency in

general have smaller fluctuations in LFP, the reduction of Var of LFP can occur in critical region even when constraining to the trials that preserve the Cri modes in pre- and post-stimulus periods (S4B Fig), and also in supercritical region (Fig 2C). It can also occur when extra stimuli modulate the chaotic attractor with Cri modes (Fig 2B) or modulate the periodic attractor (Fig 2C). In all, the synaptic decay time τ_d^I together with the input strength r_{in} induce a codimension-2 bifurcation that governs the dynamics of the network.

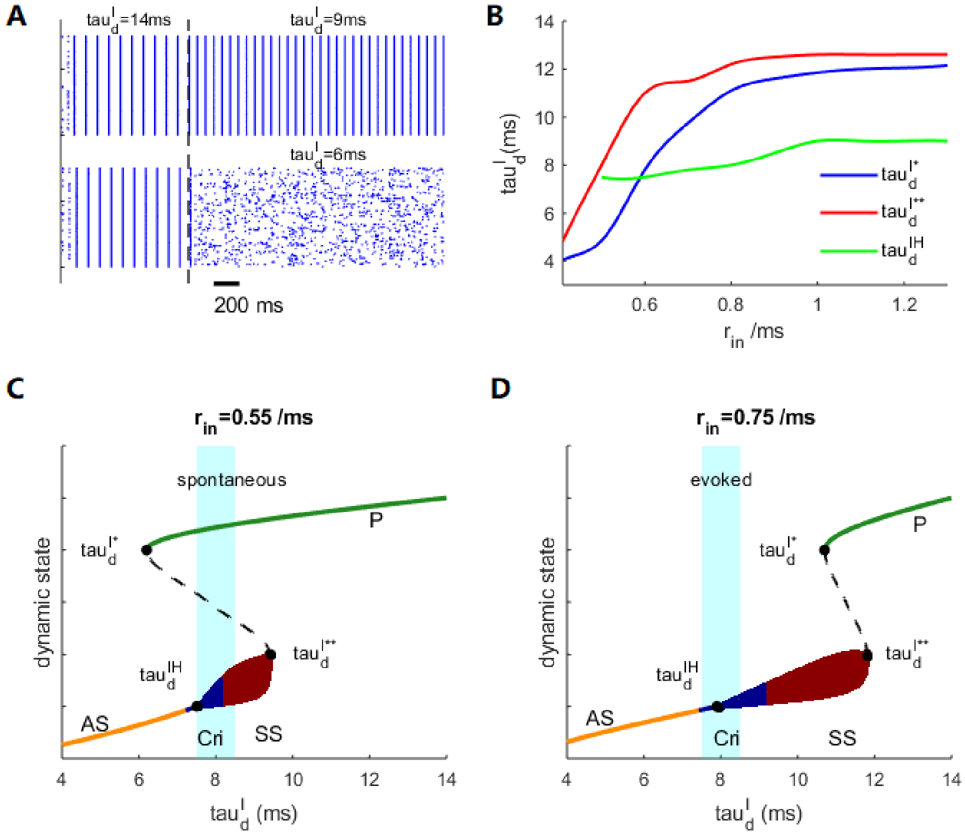

Fig a. The global deterministic dynamics of the network at spontaneous and evoked states. (A) Two examples of network simulation. In the upper case, τ_d^I is changed from 14 to 9 ms and the network maintains P mode (but with higher frequency). In the lower case, τ_d^I is changed from 14 to 6 ms and the network undergoes a transition from P mode to chaotic mode. Here, $r_{in} = 0.6/ms$. **(B)** The value of critical parameters τ_d^{I*} , τ_d^{I**} , τ_d^{IH} versus input strength r_{in}. **(C)** The heuristic bifurcation diagram of the network dynamics at $r_{in} = 0.55/ms$. The lower branch of the curve represents the chaotic attractor, which can be further divided into AS, Cri and SS modes. The upper branch represents the periodic attractor (P mode). Different modes are labeled by different color. **(D)** Same as **(C)** but for higher input strength $r_{in} = 0.75/ms$.
